# A profile of patients’ and doctors’ perceptions, acceptance, and utilization of e-health in a deprived region in southwestern China

**DOI:** 10.1371/journal.pdig.0000238

**Published:** 2023-04-25

**Authors:** Xuechen Xiong, Li Luo, Shuai Zhou, Victor Jing Li, Yinan Zhou, Zhaohua Huo

**Affiliations:** 1 School of Public Health, Fudan University, China; 2 Institute of Future Cities, The Chinese University of Hong Kong, Hong Kong, China; 3 Ruijin Hospital, Shanghai Jiao Tong University School of Medicine, Shanghai, China; 4 JC School of Public Health and Primary Care, The Chinese University of Hong Kong, Hong Kong, China; University of Bayreuth: Universitat Bayreuth, GERMANY

## Abstract

**Background:**

E-health has the potential to promote health accessibility, performance and cost-saving. However, the adoption and penetration of e-health in underprivileged areas remains insufficient. We aim to investigate patients’ and doctors’ perception, acceptance, and utilization of e-health in a rural, spatially isolated and poverty-stricken county in southwestern China.

**Methods:**

A retrospective analysis based on a cross-sectional survey of patients and doctors in 2016 was conducted. Participants were recruited through convenience and purposive sampling, and questionnaires were self-designed and validated by investigators. The utilization, intention to use and preference of four e-health services were evaluated, including e-appointment, e-consultation, online drug purchase, and telemedicine. Predictors of utilization and intention to use e-health services were investigated by multivariable logistic regression.

**Results:**

A total of 485 patients were included. The utilization rate of any type of e-health services was 29.9%, ranging from 6% in telemedicine to 18% in e-consultation. Additionally, 13.9%-30.3% of respondents as non-users revealed their willingness to use such services. Users and potential users of e-health services were inclined to specialized care from county, city or province hospitals, and they were most concerned with the quality, ease of use and price of e-health service. Patients’ utilization and intention to use e-health could be associated with education and income level, cohabitants, working location, previous medical utilization, and access to digital device and internet. There remained 53.9%-78.3% of respondents reluctant to use e-health services, mainly due to perceived inability to use them. Of 212 doctors, 58% and 28% had provided online consultation and telemedicine before, and over 80% of county-hospital doctors (including actual providers) indicated their willingness to provide such services. Reliability, quality and ease of use were doctors’ major concerns regarding e-health. Doctors’ actual provision of e-health was predicted by their professional title, number of years in work, satisfaction with the wage incentive system, and self-rated health. Nevertheless, their willingness to adopt was only associated with the possession of smartphone.

**Conclusions:**

E-health is still in its infancy in western and rural China, where health resources are most scarce, and where e-health could prove most beneficial. Our study reveals the wide gaps between patients’ low usage and their certain willingness to use e-health, as well as gaps between patients’ moderate attention to use and physician’s high preparedness to adopt e-health. Patients’ and doctors’ perceptions, needs, expectations, and concerns should be recognized and considered to promote the development of e-health in these underprivileged regions.

## Background

E-health covers a range of applications of information and communication technology (ICT) in healthcare practice, including information seeking, sharing and transmission, health education, medical consultation, telemedicine, behavioural intervention, disease management, as well as other convenience services (e.g., bill pay, medicine purchase, medical appointment) [1,2]. E-health exhibits high potential for reducing health inequities, improving efficiencies and quality, and saving healthcare costs. In the past decades, accompanied by the rapid development of ICT worldwide, e-health have expanded greatly and been applied in many different areas, including telehealth (e.g., teleradiology, telepathology, tele-dermatology, telepsychiatry), mobile health (e.g., emergency, treatment adherence, health promotion, health monitoring), eLearning, electronic health records and big data [3]. The delivery of healthcare through telephone, e-mail, internet, video conferencing and mobile applications, has proven effective in the treatment and management of different diseases, behaviour changes, and medication adherence [4–6]. In the era of the COVID-19 pandemic, e-health has also demonstrated its worth in mitigating the negative effects of infection control and social distancing on healthcare delivery [7]. Its success suggests the prominence in the future development of health systems.

Imbalance in social and economic development is widespread in China, across different regions and rural and urban areas. Disparities in health resources and outcomes are also prominent in China, which could be attributed to the individual socioeconomic status, geographic variation, rural-urban gap, and community construction [8]. To narrow these health disparities, telemedicine and e-health service have been proposed and developed for decades in China. A survey in 2017 revealed that 93% of 161 tertiary hospitals in China have carried out telemedicine services in business-to-business modalities [9]. The most common hospital-oriented telehealth services were teleconsultation, remote education, tele-diagnosis and tele-electrocardiography [9]. Meanwhile, over 130 internet hospitals, a new model and platform to integrate online and offline access for medical institutions, providing services of online appointment, teleconsultation, remote treatment and prescription, have been registered in 25 of 34 provinces or province-level municipalities in China [10]. Through internet hospitals, convenience services (registration, payment, report query), online health consultation and follow-up, telemedicine, drug distribution are available for patients. Even in rural areas of central and western China, over 58% of township health centers have set up telemedicine systems and are delivering relevant services [11]. Despite the rapid growth of e-health in the past decades, especially from the supply side, the development of e-health in China is far from enough. For example, although there are numerous studies in mHealth application, these initiatives are still concentrated on health education and behavior change through text messaging, and their evaluations are insufficient and inadequate [12]. From the consumer perspective, e-health utilization was also limited in China. According to a national survey in 2016–2017, the prevalence of online searches for health information only reached 33%, and the prevalence of other e-health behaviours (e.g., consultation, drug prescription, making appointment) was lower than 10% [13]. Low involvement of patients in e-health development can threaten its potential to promote health equity and outcomes.

In addition, development of e-health services in China requires further investigations regarding the following aspects. First, the organization and contents of e-health services vary significantly across different facilities, systems and regions. An explicit and sustainable pathway and standard to address issues on information management, health workforce, leadership and governance, and connection between online and offline service, is lacking [10]. Second, the perception and utilization of e-health services in rural and remote areas remain unclear in China, where e-health and telehealth services ought to hold the most potential. Existing studies were mainly conducted among unbalanced populations, such as phone- or internet-users, well-educated or high-income people, citizens living in developed regions, or patients in high-level heath facilities [14,15]. Rural and impoverished residents in low socio-economic status have more barriers to access telehealth, due to the geographical isolation, limited resources, lower e-health literacy, unreliable power and ICT capacity, and high costs for living [16–18]. They may also exhibit different perceptions of e-health or telehealth. For example, a survey in Montana in the United States revealed that only 5% of respondents in rural areas accepted the use of telemedicine, whereas 43% were unequivocally averse to it [19]. The situation of and the reasons behind low intention and utilization of telehealth in these regions and populations required further studies. Lastly, physicians’ attitudes and perceptions are also important to promote the delivery of e-health services, especially at primary care level in rural districts [20,21]. This study aims to investigate the perception, acceptance, and utilization of e-health from the perspectives of both patients and practitioners in an underprivileged and rural region in southwestern China.

## Method

### Study design and participants

We conducted a cross-sectional survey in a rural poverty-stricken county in Yunnan Province in 2016. Yunnan province locates in the southwest of China and is characterized by mountainous areas and plateaus. The population in Yunnan province consists of 50% rural residents, and the health professional per 1,000 residents ranks the 8^th^-least among 31 provinces, cities and autonomous regions in China. The penetration rate of mobile phones in Yunnan province is also relatively low at around 110 per 100 people, ranking the 7^th^-least province in China [22]. The selected county in our study had a high proportion (94%) of mountainous areas, and 74% of population were rural residents. The per capital gross domestic product (GDP) of this county only reaches US$5,600 (US$1 = CNY6.25) in 2020, much lower than the average level of Yunnan province (US$8,100) and China (US$10,500). To capture the full view of acceptance and utilization of e-health service from demand and supply sides, we conducted surveys separately for users and doctors, who were: 1) aged 16 and above; 2) living in the country for more than 6 months; 3) capable of understanding and responding to the survey questions in Chinese language (mandarin or local dialect). We refer these participants as potential or actual users of e-health as “patient” in our study. We calculated a sample size of 385 to have a confidence level of 95% that the true value is within ±5% of the surveyed value.

We employed a convenience and purposive sampling method to recruit our sample. From the user side, participants were recruited from all three county-level hospitals in this region, and the fairs in one town and one village. Our investigators walked around the hospitals and fairs, randomly encountered patients or residents on that day, informed them the aim of study, and invited them to complete the survey. Posters with brief introduction of the study were also put up to promote participation. People who agreed to join the survey were then interviewed by our investigators. From the provider side, doctors were recruited from the county-level hospitals, township health centers and village clinics. An invitation letter alongside a web link of questionnaire was sent to each on-duty medical professional in the county-level hospital on a specific day. They self-completed and submitted the online survey. For doctors from township health centers and village clinics, they were gathered through a training workshop held in the county hospital. At the end of meeting, they received the invitation letter and questionnaire, completed, and returned it to research staffs. The data collection lasted 5 days in August 2016. In total, 517 patients and 212 doctors were recruited and completed the survey.

The study complied with the Declaration of Helsinki and was approved by the Clinical Research Ethics Committees of Jiao Tong University. Before interview, participants were informed the objective and contents of this survey, by our investigators face-to-face or through the online survey platform. Verbal consents were obtained and witnessed under the supervision of the project manager and the administration staffs of hospitals. All data collected were anonymous and did not involve any individual-identifiable information.

### Questionnaire

The survey questionnaire was self-designed, structured, and close-ended (S1 Appendix), based on a review of previous studies on e-health [23–25]. We focused on four major types of ICT applications in healthcare provision in the context of China: e-appointment (definition: to make an electronic or web-based medical appointment through phone call, official website, mobile apps or network platform [26]), e-consultation (definition: to acquire a virtual or remote consultation or medical service from medical staffs, through phone call, text message, live chat, real-time video conference [27]), online drug purchase (definition: to buy generic or prescription medicines, with or without doctors’ prescription, via online hospital pharmacy, online retailers or e-commerce platform [28]), and telemedicine (definition: to deliver virtual or remote care to patients at a distance, including diagnosis, therapies, drug prescription and follow-up, based on telecommunication infrastructure like audio and video synchronizations, and through business-to-business or business-to-consumers models [29]). The surveys for patients and doctors were divided into three sections: 1) demographics; 2) use of digital device and internet; and 3) utilization, experience, and intention to use e-health services. The questionnaire was reviewed by a small group of experts (two professors and two clinical doctors) and pilot-tested by laymen (four graduate students and ten patients) before adoption. Minor revisions were made accordingly to improve the legibility, appropriateness, and word accuracy of it.

### Data collection and management

Data collection was completed by a research team, including one assistant professor and eleven postgraduate, graduate, and undergraduate students. Undergraduate students were mainly recruited from the local university (Dali University). They shared same living environment and language with people in the investigated area, and helped to translate and ask the questions in local dialect. All investigators possessed educational background of medicine, public health, psychological or related specialties, and were trained before the commencement of study. In the patient survey, investigators approached potential participants, administered paper-based questionnaire, and conducted face-to-face interviews. In the doctor survey, with the assistance of hospital administrators, investigators distributed invitation letters and paper- or web-based questionnaire to medical staffs, explained the survey and questions, and collected and double-checked the self-completed questionnaire by doctors. Two investigators (Z.H and S.Z) independently entered data into an electronic database (Epidata v3.1, Odense, Denmark) and crosschecked the quality of data. Missing data, error information, and incomplete questionnaire were first reviewed with the principal investigator, and would be rectified, marked as missing data (for single items), or invalid questionnaire (for separate parts), accordingly.

### Data analysis

**Fig 1:**
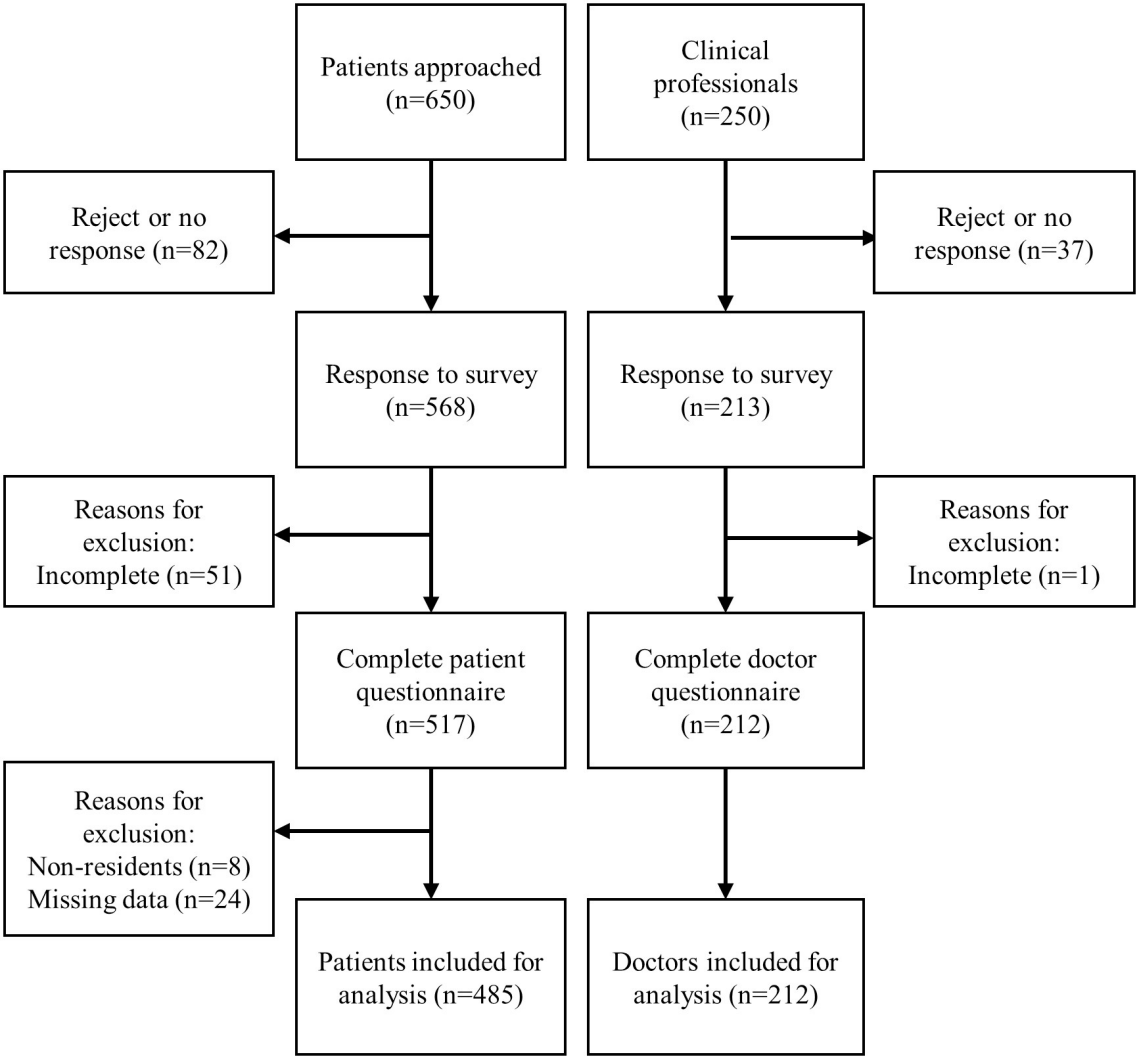
Recruitment flow.

Complete data were used for analysis, and no imputation was adopted for missing data. Eventually, 485 patients and 212 doctors were included in our analysis (Fig 1). Analyses were performed based on types of e-health service and different stakeholders (patients and doctors). According to the experience in e-health service, participants were categorized into actual users who have utilized the service before, potential users who were willing to use it, and people who were reluctant to use it. Descriptive analysis was performed of basic characteristics of participants, their usage of mobile devices and internet, and utilization and intention to use e-health services. Differences between sample subgroups were examined by Chi-square test, analysis of variance, or t-test. Correlates between factors and utilization and intention to use e-health service were first assessed by univariate analysis and crude odd ratios. Adjusted OR (aOR) and its 95% confidence interval (CI) were then estimated by multivariable logistic regression. Potential associated factors included sex, age, education, occupation, working status, residence location, cohabitant, income, medical visit in past 2 weeks, and accessibility to digital device and internet. In sensitivity analysis, considering the potential selection bias brought by convenience sampling, generalized raking procedure was performed to weight our patient sample, referring to the gender, age, and education of local population [30]. Rates of utilization and intention to use were re-estimated after sample weighting. In addition, considering that patients were recruited from different sources (hospital, town or village). We also excluded those recruited from village or town from analysis to examine its impacts on e-health utilization rates. All analyses were performed in SPSS version 21.0 (IBM Corp., Armonk, NY), and a significance level of P<0.05 was set for statistical tests.

## Results

### Participant characteristics

The basic characteristics of patients and doctors are presented in Tables 1 and 2, respectively. Among 485 patients, 64.1% were female, and the mean age was 37.4 years. Most respondents lived in rural area (80.8%), had an educational level of secondary school or lower (65.1%), and had an annual household income lower than US$4,800 (57.5%). The utilization rate of medical service in the past 2 weeks was 60.6%. A total of 342 respondents (70.5%) owned a smartphone, and 275 (56.7%) and 202 (41.6%) were internet portals and video-chat users, respectively. Only 183 (37.7%) respondents owned a personal computer, and less than 30% were equipped with network access and web camera.

**Table 1:** Basic characteristics of patients.

Characteristics, n (%)	Missing	Total	%
Total number		485	100
Recruitment location	0 (0)		
Hospitals		410	84.5
Town/Village		75	15.5
Gender	0 (0)		
Female		311	64.1
Male		174	35.9
Age group	1 (0.2)		
16–30		199	41.1
31–45		142	29.3
46–60		101	20.9
>60		42	8.7
Education level	4 (0.8)		
None or primary		172	35.8
Junior high		141	29.3
Senior high or equivalent		95	19.8
College & Higher		73	15.2
Occupation	4 (0.8)		
Farmer		246	51.1
Public or private sector		55	11.4
Freelancer or other work		91	18.9
Student, unemployed or retired		89	18.5
Working outside	0 (0)		
No		413	85.2
Yes		72	14.8
Residence	0 (0)		
Township		93	19.2
Rural		392	80.8
Cohabitant	21 (4.3)		
≤3		99	21.3
4–5		252	54.3
≥6		113	24.4
Annual household income	0 (0)		
<US$3,200		182	37.5
US$3,200–4,799		97	20.0
US$4,800–7,999		82	16.9
≥US$8,000		124	25.6
Service utilization	0 (0)		
Any service in past 2 weeks (yes)		294	60.6
Outpatient in past 2 weeks (yes)		109	22.5
Inpatient in past 2 weeks (yes)		201	41.4
Mobile device	0 (0)		
Own a smartphone (yes)		342	70.5
Access to network (yes)		275	56.7
Video-chat user (yes)		202	41.6
Usage of computer	0 (0)		
Own a personal computer (yes)		183	37.7
Access to network (yes)		161	33.2
With a web cam (yes)		100	20.6

**Table 2:** Basic characteristics of doctors.

Characteristics, n (%)	Missing	Total	Hospital level		Difference, P-value^a^
			County	Village	
Total number		212 (100)	153 (100)	59 (100)	
Gender	0 (0)				
Female		166 (78.3)	125 (81.7)	41 (69.5)	0.053
Male		46 (21.7)	28 (18.3)	18 (30.5)	
Age group	20 (10.4)				
18–30		110 (57.3)	101 (73.2)	9 (16.7)	<0.001
31–40		51 (26.6)	25 (18.1)	26 (48.1)	
>40		31 (16.1)	12 (8.7)	19 (35.2)	
Education level	0 (0)				
Secondary school		66 (31.1)	30 (19.6)	36 (61.0)	<0.001
Junior college		92 (43.4)	76 (49.7)	16 (27.1)	
Bachelor & Higher		54 (25.5)	47 (30.7)	7 (11.9)	
Professional rank	0 (0)				
Junior grade		158 (74.5)	121 (79.1)	37 (62.7)	0.014
Medium grade		54 (25.5)	32 (20.9)	22 (37.3)	
Working year	3 (1.4)				
0–3		74 (35.4)	65 (42.5)	9 (16.1)	<0.001
4–10		71 (34.0)	58 (37.9)	13 (23.2)	
11–20		33 (15.8)	15 (9.8)	18 (32.1)	
>20		31 (14.8)	15 (9.8)	16 (28.6)	
Actual monthly income	0 (0)				
<US$160		18 (8.5)	0 (0.0)	18 (30.5)	<0.001
US$160–319		75 (35.4)	52 (34.0)	23 (39.0)	
≥US$320–959		115 (54.2)	97 (63.4)	18 (30.5)	
≥US$960		4 (1.9)	4 (2.6)	0 (0.0)	
Expected monthly income	0 (0)				
US$160–960		102 (48.1)	53 (34.6)	49 (83.1)	<0.001
US$960–1,600		91 (42.9)	81 (52.9)	10 (16.9)	
≥US$1,600		19 (9.0)	19 (12.4)	0 (0.0)	
Working environment	0 (0)				
Time for communication (yes)		121 (57.1)	92 (60.1)	29 (49.2)	0.148
ICT promotes my work (yes)		123 (58.0)	89 (58.2)	34 (57.6)	0.943
Adequate income system (yes)		79 (37.3)	53 (34.6)	26 (44.1)	0.203
Too much workload (yes)		124 (58.5)	85 (55.6)	39 (66.1)	0.163
Being respected (yes)		100 (47.2)	59 (38.6)	41 (69.5)	<0.001
Self-rated health	0 (0)				
Enough time for sleep (yes)		47 (22.2)	37 (24.2)	10 (16.9)	0.256
Enough time for exercise (yes)		45 (21.2)	34 (22.2)	11 (18.6)	0.568
Well physical health (yes)		100 (47.2)	73 (47.7)	27 (45.8)	0.799
Well mental health (yes)		117 (55.2)	87 (56.9)	30 (50.8)	0.430
Usage of mobile device	0 (0)				
Own a smartphone (yes)		195 (92.0)	144 (94.1)	51 (86.4)	0.065
Access to network (yes)		184 (86.8)	140 (91.5)	44 (74.6)	0.001
Video-chat user (yes)		131 (61.8)	96 (62.7)	35 (59.3)	0.646
Usage of computer	0 (0)				
Computer at workplace (yes)		199 (93.9)	149 (97.4)	50 (84.7)	0.001
Access to network (yes)		111 (52.4)	70 (45.8)	41 (69.5)	0.002
With a web cam (yes)		28 (13.2)	24 (15.7)	4 (6.8)	0.086

^a^. Differences between groups were tested by χ^2^ tests.

Among 212 doctors, 78.3% were females, and the mean age was 31.2 years. All of the surveyed doctors had an education level of secondary school or higher. Most of them had ten or lower years in work (69.4%) and had a monthly income lower than US$960 (98.1%). Over a half of doctors were satisfied with the time for patient communication and (57.1%) application of ICT in their facilities (58.0%). Nevertheless, less than a half of them were satisfied with the wage incentive system (37.3%), daily workload (41.5%), being respected by the society (47.2%), time for sleep and exercise (<25%), and their own physical health (47.2%). Most clinicians owned a smartphone or were equipped with an office computer (>90%), and they had access to internet through mobile phone (86.8%) or computer (52.4%). In addition, doctors from village health facilities, compared to those from county-level hospitals, were older, less educated, working for longer years but with lower income and lower usage of computer and internet.

### Patients’ utilization and perception of e-health service

#### Utilization and experience of e-health service

**Fig 2:**
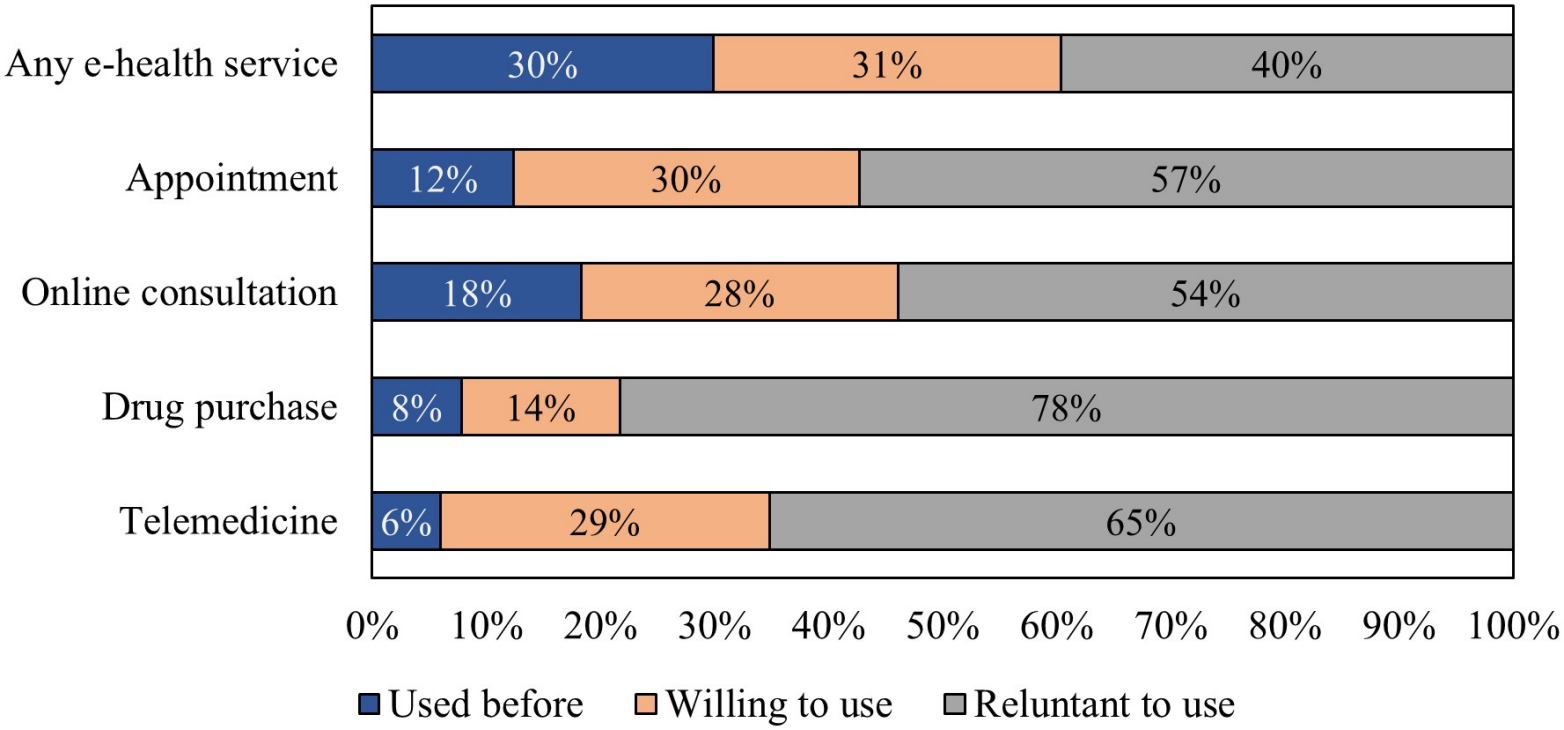
Patients’ utilization of and intention to use e-health service.

**Fig 3:**
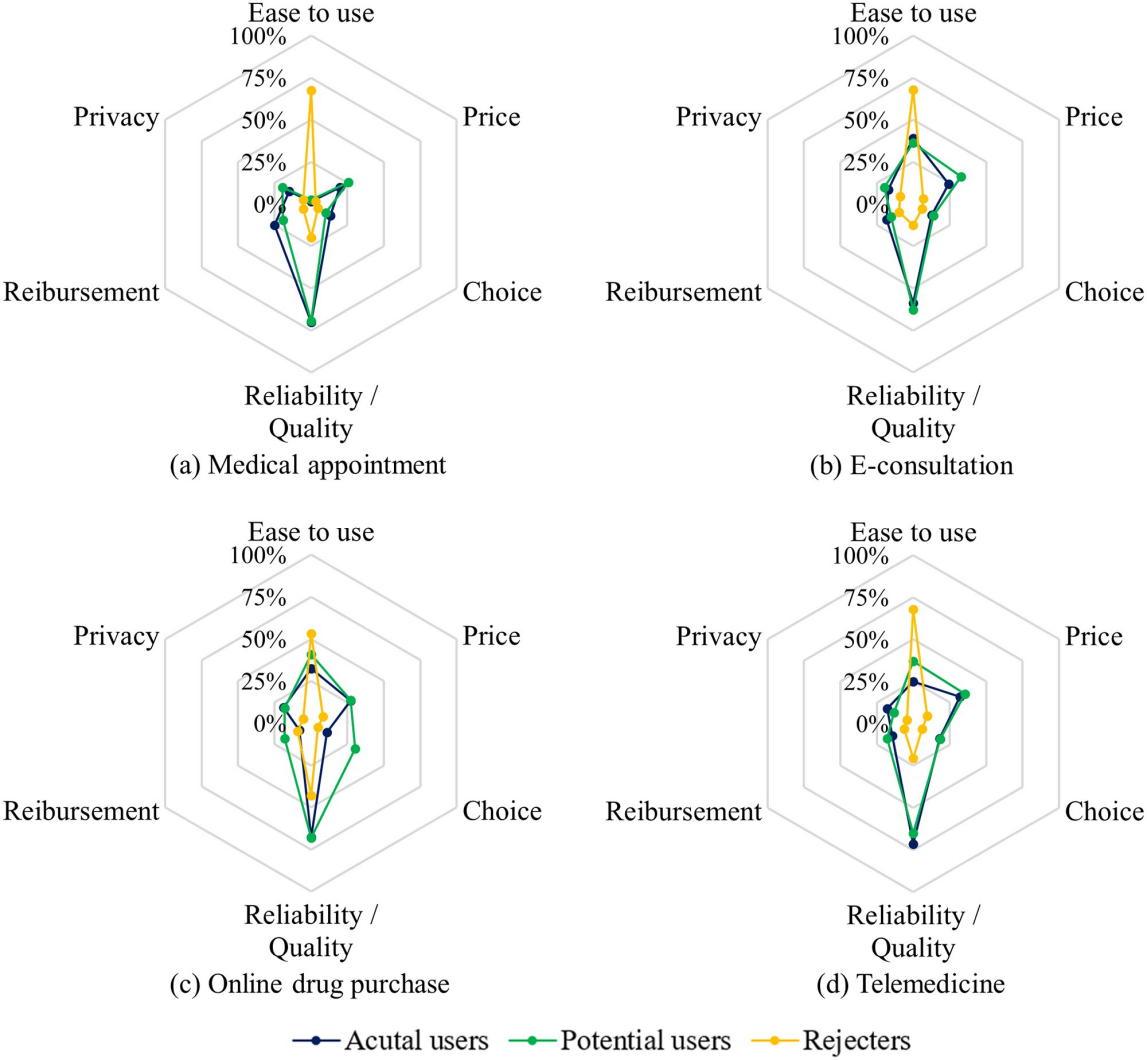
Concerned factors associated with utilization and intention to use e-health service among patients.

A total of 145 (29.9%) respondents had utilized at least one type of e-health service before, while 148 (30.5%) indicated willingness to use (Fig 2). The detailed responses of patients to each question are shown in S2 Appendix. Regarding e-appointments, 12.4% respondents had scheduled an e-appointment before, mainly through an official website and hotline (63.8%). The factors of most concern by current users were reliability (70.0%), reimbursement policy (25.0%) and price (20.0%) (Fig 3). For e-consultations, 18.3% respondents had utilized such service before, mainly for chronic diseases (57.7%) and with no charge (77.4%). The factors of most concern were reliability (58.6%), ease of use (39.1%) and price (24.1%). For e-purchase of medicines, only 7.8% respondents had utilized it before, mainly for generic drugs (51.6%). The most concerning factors of e-purchase were quality (67.6%), ease of use (32.4%) and price (27.0%). For telemedicine, only 6.0% respondents had utilized it before, mainly for severe health problems (e.g., surgery, tumor) (53.6%). Telemedicine users were most concerned with the quality (71.4%), price (32.1%), and ease of use (25.0%) of service. Finally, we found the major motivations of consumers to use different e-health services were the physical inaccessibility of services and reference from doctors or close persons, and most e-health services involved higher levels of hospitals (secondary or tertiary care) and medical specialists. Over 60% of e-health users stated the price and performance of e-health services was comparable to that of on-site modes, except for telemedicine. Over half of telemedicine users were unclear about the performance of telemedicine or reported medical disputes of it.

#### Willingness to use e-health service

For e-appointment, 147 (30.5%) respondents indicated willingness to use it (Fig 2). Potential users preferred to make e-appointment through an official website or hotline (84.5%) and with a specialist (66.4%). For e-consultation, 132 (27.8%) respondents exhibited a willingness to use it, mainly for the diagnosis and treatment of chronic or severe diseases (>60%). For online drug purchase, only 66 (13.9%) respondents were willing to use it. For telemedicine, 135 (28.8%) respondents were willing to use the service, mainly for severe illness (69.6%). Potential users of telemedicine also expected a doctor accompanying them and a specialist from secondary or tertiary care hospitals at a remote site (>60%). Finally, the major motivations to use different e-health services among potential users were unavailability and inconvenience for high-quality healthcare service (>50%). Potential users of different e-health services were most concerned with the reliability, quality, price, and ease of use of service; while the main reasons for those reluctant to use were inability to use and concerns about reliability and quality (Fig 3). Regarding price, over half of potential users expected the charge of e-health service same as or lower than on-site care.

#### Correlation analysis

Multivariable analyses between different factors and patients’ utilization and intention to use e-health service are presented in S3 Appendix. Results indicates those with college degree (vs primary school or lower) were 15 times more likely to purchase drugs online (aOR: 15.82, 95%CI [2.29, 109.07], P = 0.005) and 2 to 4 times more willing to use e-appointment (aOR: 3.28 [1.17, 9.21], P = 0.009) and online drug purchase (aOR: 4.71 [1.48, 14.95], P = 0.009). People living with over three cohabitants (vs <3 cohabitants) used more e-purchasing of drug (aOR 3.73–5.97 [1.16, 22.83], P<0.05), and they were also more willing to use it (aOR 2.72–2.80 [1.19, 6.60], P<0.05). Compared to farmers, the “other” group (mainly composed of students) showed 1.4 times more willingness to use telemedicine (aOR: 2.40 [1.17, 4.92], P = 0.017). People going out work utilized more e-appointment (aOR: 2.32 [1.11, 4.84], P = 0.024) and online medicine purchasing services (aOR: 2.69 [1.08, 6.73], P = 0.034), and they were also more willing to use e-appointment (aOR: 2.01 [1.11, 3.66], P = 0.024) and online consultation (aOR: 2.43 [1.30, 4.53], P = 0.005). People with higher annual household income (>US$3,200 vs <US$3,200) was 1 to 4 times more willing to use online consultation (aOR 1.91–1.94 [1.02, 3.64], P<0.050), online drug purchase (aOR 2.30 [1.14, 4.64], P = 0.020), and telemedicine (aOR 1.90–2.56 [1.02, 4.89] P<0.050). People hospitalized in the past 2 weeks inversely used less online drug purchase (aOR: 0.23 [0.07, 0.69], P = 0.009]). Finally, people owning a smartphone were more willing to use e-appointment (aOR: 3.98 [2.08, 7.60], P<0.001), online consultation (aOR: 2.56 [1.36, 4.81], P = 0.004), and telemedicine services (aOR: 2.04 [1.06, 3.92], P = 0.033), while those owning a personal computer utilized more e-appointments (aOR: 2.76 [1.22, 6.25], P = 0.015) and were more willing to use online consultation (aOR: 1.90 [1.07, 3.37], P = 0.029).

### Doctors’ provision and perception of e-health service

#### Provision and experience of e-health service

**Fig 4:**
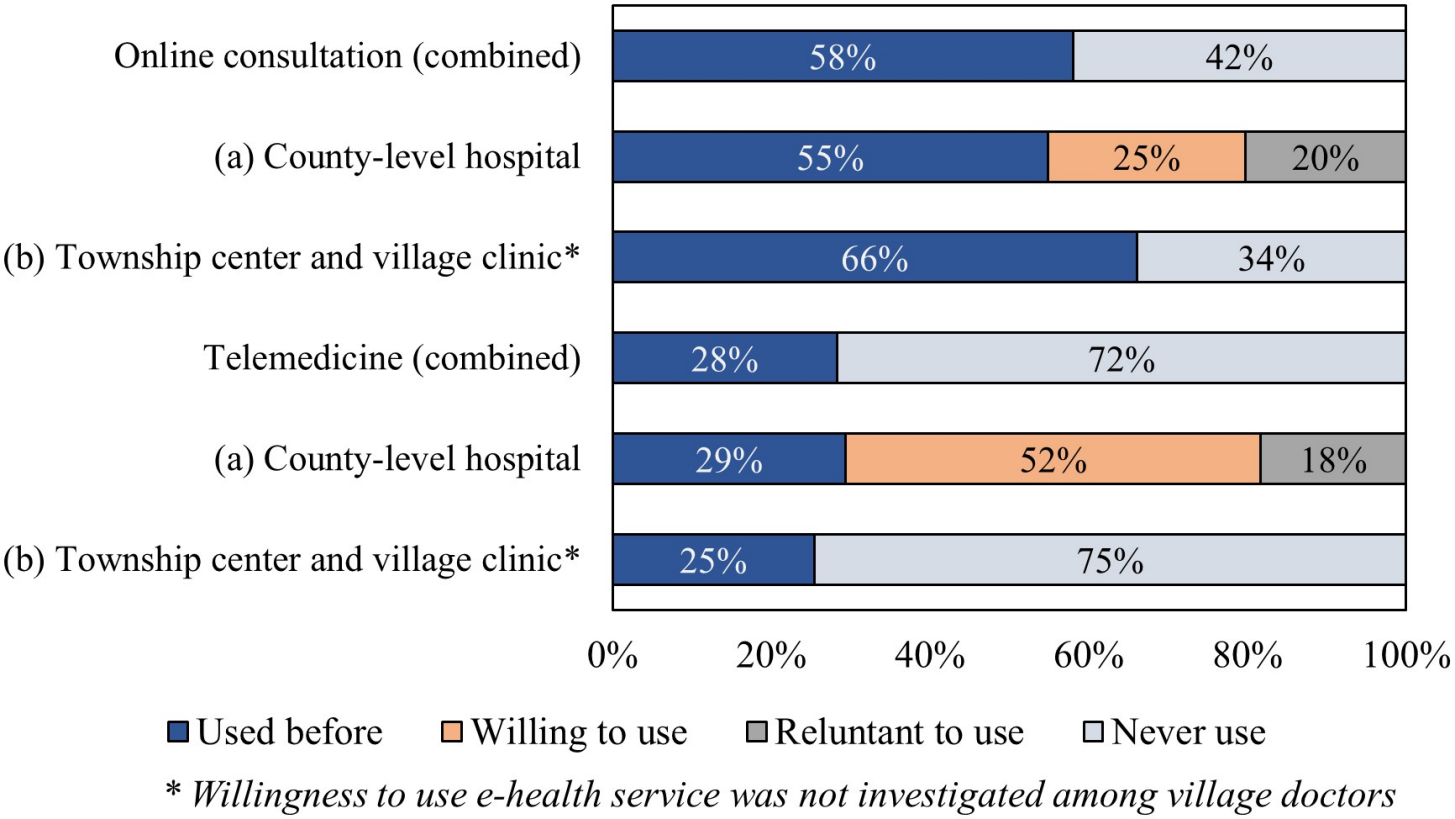
Doctors’ provision of and intention to use e-health service.

**Fig 5:**
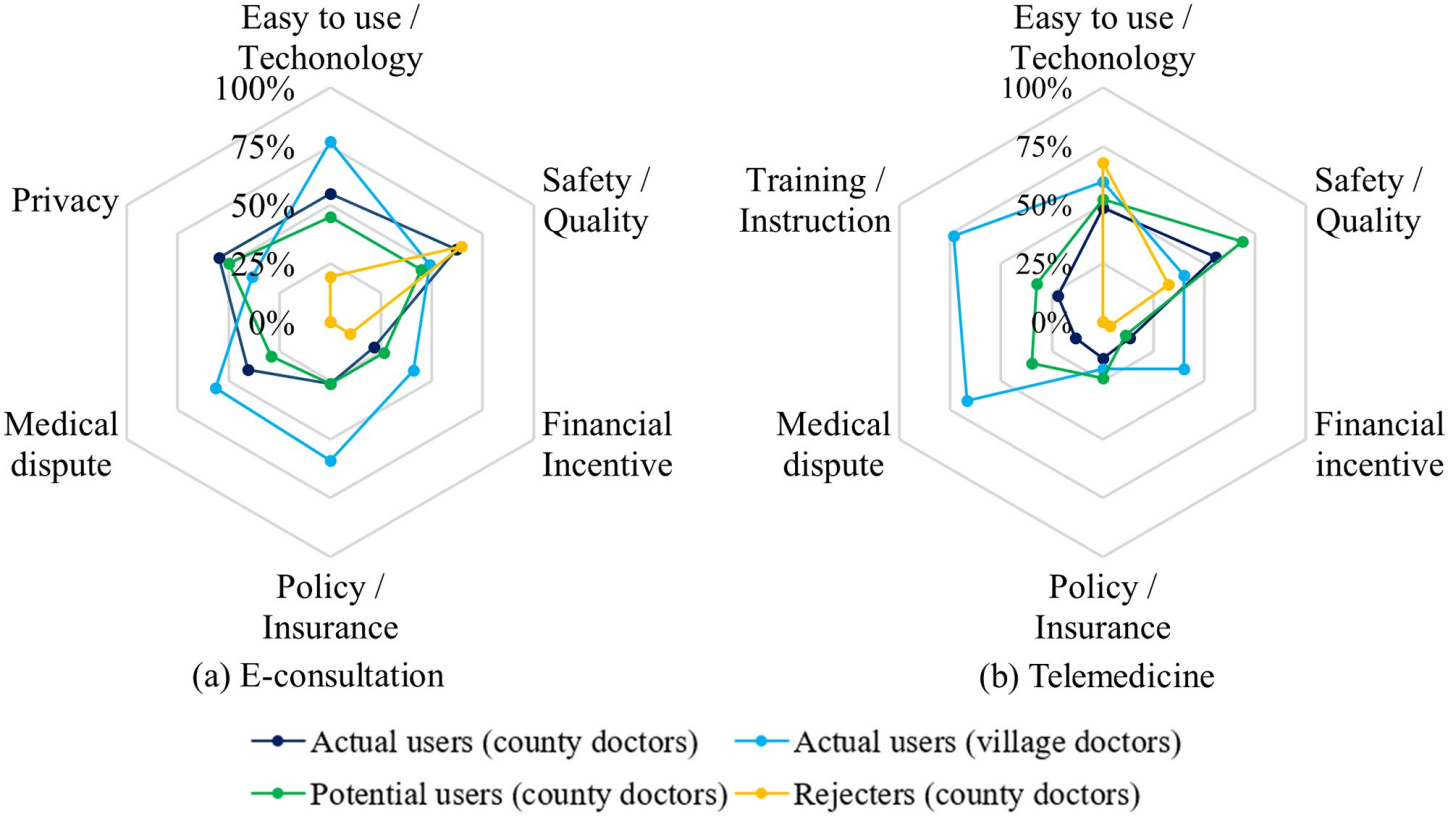
Concerned factors associated with provision and intention to adopt e-health service among doctors.

Among 212 doctors, 123 (58.0%) and 60 (28.3%) had provided online consultation and telemedicine before, with no significant differences between county-level and village doctors (Fig 4). Doctors provided e-health services mainly due to their work needs (>65%) and patients’ requests (>40%). The detailed responses of doctors to the survey are shown in S4 Appendix. Doctors mainly provided e-consultation via phone call (>90%), WeChat (an instant messaging application like WhatsApp, >45%) or text messaging (>45%). Consultations mainly involved chronic disease (>80%), and the diagnosis (>70%) and treatment (>55%) of diseases. Although 90% of doctors did not get paid from e-consultation, 77% of them expected a payment for it. Regarding telemedicine, doctors adopted it for both chronic and severe illness. Instructors of telemedicine at remote sides included doctors from different levels of institutions (city hospital, county hospital or village health center). Over 70% of doctors undertook medical liability during such services and over 80% of them were satisfied with the performance of them. The major concerns of actual providers regarding e-consultation and telemedicine were safety and quality of service, ease of use, technological preparedness, and skills training (Fig 5). Village doctors were further concerned about the policy or insurance support and medical disputes encountered.

#### Intention to provide e-health service

The intention to provide e-health services was only available for county-hospital doctors. Regarding online consultation, 38 (24.8%) of 153 doctors indicated willingness to deliver it (Fig 4). They preferred to deliver e-consultation on the treatment and medication use of chronic disease, via official website, phone call or WeChat. Over 80% of doctors also expected a payment for e-consultation. For telemedicine, 80 (52.3%) doctors were willing to adopt it for chronic or severe diseases. Over 70% of them agreed that there should be a face-to-face contact with patients before service, and there should be a confirmation of patient identification and consent, access to patients’ medical history, and a doctor at a patient’s side during service. Most of doctors recognized the advantages of telemedicine in promoting service accessibility (72.5%), health outcomes (65.0%) and medical skills (52.5%), and they considered the necessity for specific regulations and standards for telemedicine (>80%). Only 56% of potential providers considered physical examination was necessary for telemedicine, and 60% were unclear about the performance of telemedicine when compared to routine care. The major concerns of potential providers of both e-consultation and telemedicine were safety and quality of service, and ease of use and technological preparedness (Fig 5). Doctors were also concerned about the privacy of e-consultation, as well as the medical disputes encountered in telemedicine. Finally, the main reasons for doctors’ reluctance to adopt e-health were inability to use, concern about reliability and quality, and over workload.

#### Correlation analysis

Multivariable analyses between factors and doctors’ provision and intention to adopt e-health service are presented in S5 Appendix. Results showed that doctors with a higher grade of professional title (medium-grade vs junior-grade) were 1.3 times more likely to deliver telemedicine services (aOR: 2.28 [1.00, 5.20] P = 0.050), but not for online consultations. Doctors with 4 to 10 years in work inversely provided (vs <4 years) less telemedicine service (aOR: 0.40 [0.17, 0.96], P = 0.039). Doctors satisfied with the current income incentive system also provided more telemedicine service than those not satisfied (aOR: 3.95 [2.03, 7.71], P = 0.001). Doctors’ perception of heavy workload and self-rated physical health did not show significant impacts on the provision and intention to adopt e-health services. However, better self-rated mental health was associated with more provision of online consultation (aOR: 3.27 [1.17, 9.18], P = 0.024). Finally, doctors possessing a smartphone were 5–7 times more willing to adopt online consultation (aOR: 7.55 [1.30, 44.00], P = 0.025) and telemedicine (aOR: 5.89 [1.05, 32.98], P = 0.044), compared to those without a smartphone.

### Sensitivity analysis

After weighting, the sample distribution of patients was comparable to the population in local region (S6 Appendix). The re-estimated rates were slightly lower for e-health utilization (differences: -1.1% to -2.9%) and willingness to use (differences: -1.6% to -2.3%) among patients. Besides, after excluding participants recruited from towns or villages (n = 75, 15.5%) (S7 Appendix), the variations in re-estimated rates were also subtle (difference: ±2.5%).

## Discussion

### Main findings

This study provides a comprehensive view of people’s perception, acceptance, and utilization of e-health in a rural and poverty-stricken county in western China. From patients’ perspectives, around 30% of respondents had utilized e-health service before, ranging from 6% in telemedicine, 8% in online medicine purchase, 12% in e-appointment, to 18% for e-consultation. These rates are comparable to or lower compared to previous studies in China (National survey, 2016–2017: 9% in buy medicine online, 10% in doctor consultation; 10% in online appointment [13]; Patient survey in Chengdu, 2016: 28% in appointment, 10% in virtual visit [31]; General population in Hong Kong SAR, 2016–2018: 47% in e-health usage, 38% in health apps, 27% in e-appointment; 19% in doctor contact, 13% in medicine order; 2% in telehealth [2,32,33]). It should be pointed out that the relatively high utilization rates of e-appointment and e-consultation in this study could be due to our inclusion of phone calls and messages as delivery approaches of e-health. Regarding the price and performance of e-health services, most users in our study stated its equivalence as traditional care, except for telemedicine. We further identified a considerable gap in the e-health market, where around 30% of respondents as nonuser revealed their willingness to adopt them. Combining actual usage and willingness to use, the most accepted e-health service was e-consultation, followed by e-appointment, telemedicine, and online drug purchase. Potential users expected high-quality and more extensive service from e-health, including care for both chronic and severe diseases and services provided by different levels of health facilities and professionals. They also expected a same or lower price of e-health services when compared to conventional care. The major concerns of patients regarding e-health services included reliability and quality, ease of use, and price of services, consistent with the findings from previous studies [33–37]. Finally, there were still over a half of respondents reluctant to use e-health services, mainly due to their perceived inability to use, and concerns about reliability and quality of service.

From doctors’ perspectives, 58% and 28% of surveyed doctors had provided e-consultation and telemedicine before, reflecting the limited integration of ICT into care provision in this county. Phone call is still the major approach to provide e-consultation, followed by instant messaging. Most of e-consultation services were free of charge and involved chronic disease. For telemedicine in practice, it covered both chronic and severe diseases. The organization of it followed a tight connection between city-, county-, and village-level facilities, and most doctors undertook medical liability in providing such services. Most e-health providers were satisfied with the performance of telemedicine, in contrast to the perception of consumers. Regarding intention to use, doctors’ willingness to provide online consultation and telemedicine was also high (>80%), which was opposite to the low intention to use (<50%) among patients. Most doctors were inclined to provide e-consultation on chronic disease, via different channels, and with a charge. In terms of telemedicine, most doctors were willing to adopt it for both chronic and severe disease, and they acknowledged the importance of operational norms on it, including a physician alongside patient, patient identification and consent, and face-to-face contact before service. However, only half of potential providers considered physical examination necessary, and most of them were unclear about the performance of telemedicine. Reliability, quality, ease of use and technology preparedness were doctors’ major concerns on e-consultation and telemedicine. Policy support, financial incentive, training, medical disputes, and privacy could also not be neglected from doctors’ point of view. Finally, there remains around 20% of doctors unwilling to adopt e-health services, mainly due to the perceived inability to use, concerns about safety and quality, and heavy workload.

Predictors of utilization and willing to use e-health services were also investigated. Patients’ utilization and intention to use e-health services was associated with higher educational and income, family cohabitant, possession of smartphone and computer, and access to internet, which are consistent with the previous findings [13,31,32,38]. However, gender was not significant indicators in our analysis. People working outside as migrants and students also showed higher acceptance of e-health. They might be characterized by younger age and higher education level, and are expected to have higher interest and capacity to use innovative technologies and products. It is unexpected that hospitalized patients in our study were less inclined to purchase drugs online. The potential explanation is that online purchase behaviour mainly originates from patients’ needs for self-treatment for mild symptoms, and people with acute or severe symptoms were more likely to seek help from local hospitals. From provider perspectives, doctors’ provision of online consultation and telemedicine can be predicted by higher professional title, shorter years in work (kin to younger age), satisfaction with their income level, and self-rated health. Nevertheless, we found that doctors’ willingness to adopt e-health services was only affected by the possession of smartphones after controlling for other variables.

### Implications

Our findings provide insight for clinical practice, policy making and further studies of the development of e-health in less developed regions. First, ICT infrastructure and popularization were still a major barrier for e-health development in rural and remote regions. Compared to the average level in Yunnan and other regions in China [22], our study sample revealed a greater digital divide among the population, where 43% of patients still did not have access to internet, and 58% of them did not know how to use video chat by phone (even among doctors, this figure could reach high as 40%). Our analyses also reveal that access to digital devices and internet is a strong predictor for patients’ and doctors’ utilization and willingness to adopt e-health services. Previous studies have pointed out that digital divide in rural areas and deprived populations could lead to health disparities and inequities, and was a major barrier to telehealth [39,40]. Therefore, there still needs a persistent investment in the penetration and popularization of ICT and digital usage in these areas, to ensure the ubiquitous access to e-health among most vulnerable and underprivileged populations.

Second, health and technology education are necessary for patients and doctors in rural areas to promote their e-health literacy and adoption of e-health. Although a high proportion (>50%) of patients were reluctant to use e-health services from our findings, the primary reason for their refusal was their perceived inability to use the services, which is also mentioned by previous studies [41,42]. Among doctors, training and instruction is also one of the major concerns regarding the adoption of e-health. Education and training programs related to the introduction, usefulness, applicability and practice skills of e-health, should be established for both patients and doctors. Public media and social acquaintances can play an important role in this, for we found a number of e-health users in our study were motivated because of references from persons close to them (family members, friends or doctors). Third, specific attention and efforts are required for disadvantaged groups to increase their initiatives and abilities to adopt e-health, including older adults, minorities, people with low socioeconomic status or physical and cognitive impairment [43,44]. Potential solutions include multiple platforms to deliver e-health (e.g., message, phone call, website, portal device), user-friendly and user-specific design of e-health applications, support and help by practice assistants and family members, and instructions from medical staff [38,45]. Fourth, clear clinical guidelines and standards should be formulated for different types of e-health service, and integration of online and offline services should be realized so as not to excessively increase doctors’ daily workload (especially for village doctors who also take responsibility of public health missions). Medicolegal exposure, reimbursement parity, insurance policy, personal privacy, and pricing of e-health services should also be regulated to balance the interests among patients, doctors, institutions, and payers [31,46].

Finally, as a baseline analysis, this study provides an important reference for further follow-up studies on the development of e-health and telehealth in rural, remote, and poor regions, especially under the development of economy and society, the penetration of ICT technology, and the outbreak of COVID-19 pandemic.

### Strengths and limitations

This study has several strengths compared with previous studies on the topic of e-health provision and acceptance. First, it is among the few studies that focus on the development of e-health in rural, remote, and poor regions. For a long time, inhabitants in rural and remote areas had less access to health resources. They are as such most in need of e-health and telehealth services, but remain perpetually underserved and under-investigated. Our study faithfully reflects the actual needs and expectations of underprivileged grassroots and primary care providers regarding e-health services. Second, this study possesses extensive scope and depth. We identify gaps between the actual utilization and willingness to use e-health services, gaps between patients’ and doctors’ perception and expectation on e-health services, and gaps of e-health utilization and acceptance by various types of service. We also investigated patients’ and doctors’ expectations on the modality, contents, and price of different e-health services. Third, we included patients’ and doctors’ access to digital devices and internet in our survey. We highlighted the digital divide present in this remote and poor area, and its impacts on e-health utilization and intention to use. We also performed multivariable analyses to explore associations between participants’ sociodemographic factors and their utilization and willingness to use e-health services, including living in town or village, having medical visit or not, and so on, to reduce the potential confounding bias present in previous studies.

Our study, however, possesses some limitations. First, selection bias may exist due to non-random sampling. Seventy-five participants were excluded from analysis due to incomplete survey or missing data tended to be older and less educated. As a result, participants included in our study were younger and more educated than the wider population. To resolve this problem, we performed sample weighting based on population demographics. The adjustments only showed slight impacts on the rates of utilization and intention to use e-health services (dropped by 1–3%), and the main findings remain unchanged. Second, definition of “patient” in our study did not strictly confirm to those with illnesses and in need of cure. Neither can we tell healthy participants from those with diseases from our data, because we did not ask information on their medical condition or comorbidity. Instead, we refer “patient” in our study as a broader group who has the potential to use e-health in the future. Despite this limitation, we included an indicator “having a medical visit in past 2 weeks” in correlation analysis. No major associations between it and utilization and intention to use e-health were found among patients after controlling other confounders (e.g., education, income, accessibility to digital devices and internet). This may reflect the surrogate relationship between the public and patients in investigating e-health acceptance [20]. Similarly, recruitment from hospitals, towns or villages did not show significant impacts on the outcomes from our analyses.

Third, deficiencies exist in our survey design. Several potential predictors of utilization and intention to use e-health were not available from our survey, such as patients’ comorbidity and ethnicity, which may cause unobserved confounding. In particular, the selected site of our survey was composed of multiple minority groups, occupying 43% of total population. We neither investigate the awareness and accessibility of e-health services among respondents, which could be the associated factors of e-health utilization and intention to use. In addition, we did not investigate the intention to use e-health service among actual users and cannot examine the relationship between behavioural intention and actual use. We neither asked about the intention to adopt e-health service among village doctors. Finally, we did not investigate other applications of e-health and telehealth, such as information seeking, health education, mobile health, wearable devices and so on. Despite these deficiencies, we found it difficult to have in-depth conversations with participants in our study, because e-health and telehealth was still in its infancy in this region, and most patients and doctors lacked profound awareness and understanding of it.

Lastly, our study was limited by cross-sectional and retrospective analysis of a field survey undertaken five years ago. With greater integration and penetration of digital technology and ICT into daily life and society, as well as the changing social environment under COVID-19 pandemic, the awareness, perception, application and utilization of e-health among patients and doctors may develop accordingly [47–50]. Longitudinal follow-up studies are needed to track these changes [37].

## Conclusion

E-health is still in its infancy in western and rural China, where health resources are most scarce, and where e-health could prove most beneficial. Our study in a deprived region reveals the wide gaps between patients’ low usage and their certain willingness to use various types of e-health services, as well as gaps between patients’ moderate attention to use and physicians’ high preparedness to adopt them. Patients’ and doctors’ perceptions, needs, expectations, and concerns should be recognized and considered to promote the development of e-health in China’s most underprivileged regions.

## Supporting information

S1 Appendix Survey questionnaire. (DOCX)

S2 Appendix Patients’ responses to survey items by types of service and user groups. (DOCX)

S3 Appendix Univariate and multivariable analyses of factors associated with patients’ utilization and intention to adopt e-health service. (DOCX)

S4 Appendix Doctors’ responses to survey items by types of service and user groups. (DOCX)

S5 Appendix Univariate and multivariable analyses of factors associated with doctors’ provision and intention to adopt e-health service. (DOCX)

S6 Appendix Weighting patient sample by population demographics. (DOCX)

S7 Appendix Exclusion of participants recruited from towns or villages. (DOCX)
