# Supplementary material for: A profile of patients’ and doctors’ perceptions, acceptance, and utilization of e-health in a deprived region in southwestern China
Source: PLOS Digit Health. 2023 Apr 25;2(4):e0000238. doi: 10.1371/journal.pdig.0000238 (PMC10129013; doi:10.1371/journal.pdig.0000238)
Supplement: S1 Appendix — (DOCX) [file pdig.0000238.s001.docx]

# S1 Appendix. Survey questionnaire

Part I. Patients survey

| **(a) Demographics** | |
| --- | --- |
| Gender | □Male □Female |
| Age | ____years |
| Education level | □Primary school or lower □Junior high school or equivalent □Senior high school or equivalent □College / University and higher |
| Occupation | □Government or public institution □State-owned or private enterprise □Farmer □Freelancer □Retired □Student □Others (Unemployment) |
| Residence | □Town □Village |
| Migrant workers | □No □Yes |
| Insurance | □Free medical service □Social insurance for employees □Social insurance for residents □Rural cooperative medical care system □Others (Medical aids, no insurance) |
| Cohabitants | ____persons |
| Annual household income (1CNY=0.16USD) | □<CNY20,000 □CNY20,000-29,999 □CNY30,000-39,999 □CNY40,000-49,999 □≥CNY50,000 |
| Outpatient service in the previous 2 weeks | □No □Yes |
| Inpatient service in the previous 2 weeks | □No □Yes |
| **(b) Usage of digital device and internet** | |
| Owning a smartphone | □No □Yes |
| Phone access to internet | □No □Yes |
| Able to use video-chat | □No □Yes |
| Owning a personal computer | □No □Yes |
| Computer access to internet | □No □Yes |
| Computer equipped with camera | □No □Yes |
| **(c) E-appointment**  *Introduction by investigator: e-appointment allows patients to make a medical appointment without in-person visit at hospitals, through phone call, official website, mobile apps, network platform, etc.* | |
| Use e-appointment before | □No □Yes |
| (If yes) |  |
| Platform to make appointment | □Official website/hotline □Third-party platform □Other_____ |
| Hospital to make appointment | □Province-level hospital □City-level hospital □County-level hospital □Other_____ |
| Type of doctor to make appointment | □General practitioner □Specialist □Other_____ |
| Price compared to offline mode | □Same □Higher □Lower □Unclear |
| Satisfied with the service | □No □Yes □Hard to say |
| (If no) |  |
| Willing to use | □No □Yes |
| (Not willing to use) |  |
| Reason (multiple selection) | □Unable to use □High price □Limited choice □Unreliable □No insurance reimbursement □Privacy security □Not necessary □Other__________ |
| (Willing to use) |  |
| Platform preferred | □Official website/hotline □Third-party platform □Other_____ |
| Hospital preferred | □Province-level hospital □City-level hospital □County-level hospital □Any/Not specified |
| Doctor preferred | □General practitioner □Specialist □Any/Not specified |
| Expected Price compared to offline mode | □Same □Higher □Lower □Unclear |
| Most concerned factors (multiple selection) | □Ease to use □Price □Choice □Reliability □Insurance reimbursement □Privacy security □Other__________ |
| **(d) E-consultation**  *Introduction by investigator: e-consultation allows patients to acquire virtual/remote consultation or medical services with medical staffs (doctors, nurses, or specialists), through phone call, text message, live chat, real-time video conference, etc. Such services can be realized by telephone, mobile phone apps, official or third-party platform, and so on. Usually e-consultation does not involve formal medical diagnosis and drug prescription.* | |
| Use e-consultation before | □No □Yes |
| (If yes) |  |
| Motivations to use | □Inaccessibility to service □Inconvenience to face-to-face visit □Reference from others □Other_____ |
| Hospital to consult | □Province-level hospital □City-level hospital □County-level hospital □Other_____ |
| Type of doctor to consult | □General practitioner □Specialist □Other_____ |
| Disease to consult about (multiple selection) | □General symptom/Chronic disease □Severe illness (surgery/tumour) □Other______ |
| Content to consult about (multiple selection) | □Diagnosis □Treatment □Fee □Medication use □Follow-up □Other______ |
| Confirmation of doctors’ identification | □No □Yes □Unclear |
| Price of service | CNY_____ |
| Satisfied with the service | □No □Yes □Hard to say |
| (If no) |  |
| Willing to use | □No □Yes |
| (Not willing to use) |  |
| Reason (multiple selection) | □Unable to use □High price □Limited choice □Low reliability/quality □No insurance reimbursement □Privacy security □Not necessary □Other__________ |
| (Willing to use) |  |
| Motivation to use | □Inaccessibility to service □Inconvenience to face-to-face visit □Reference from others □Other_____ |
| Hospital preferred | □Province-level hospital □City-level hospital □County-level hospital □Any/Not specified |
| Doctor preferred | □General practitioner □Specialist □Any/Not specified |
| Disease preferred (multiple selection) | □General symptom/Chronic disease □Severe illness (surgery/tumour) □Other______ |
| Content preferred (multiple selection) | □Diagnosis □Treatment □Fee □Medication use □Follow-up □Other______ |
| Requirement to confirm doctor identification | □No □Yes □Unclear |
| Willing to pay | □Free □CNY1-10 □CNY10-20 □CNY20-30 □CNY>30 |
| Most concerned factors (multiple selection) | □Ease to use □Price □Choice □Reliability/Quality □Insurance reimbursement □Privacy security □Other__________ |
| **(e) Online drug purchase**  *Introduction by investigator: online drug purchase allows patients to buy generic or Rx drugs, with or without doctors’ prescription, through online hospital pharmacy, online retailers, e-commerce platform, etc.* | |
| Use e-purchase before | □No □Yes |
| (If yes) |  |
| Motivations to use | □Inaccessibility to drug □Inconvenience to buy drug □Reference from others □Other_____ |
| Type of drug to buy | □Rx drug □Generic drug □Not specified |
| Price compared to offline purchase | □Same □Higher □Lower □Unclear |
| Requirement of doctor prescription | □No □Yes □Not applicable |
| Confirmation of sale qualification | □No □Yes □Unclear |
| Satisfied with the service | □No □Yes □Hard to say |
| (If no) |  |
| Willing to use | □No □Yes |
| (Not willing to use) |  |
| Reason (multiple selection) | □Unable to use □High price □Limited choice □Low reliability/quality □No insurance reimbursement □Privacy security □Not necessary □Other__________ |
| (Willing to use) |  |
| Motivation to use | □More choices □Time saving □Reference from others □Cost saving □Other_____ |
| Expected Price compared to offline mode | □Same □Higher □Lower □Unclear |
| Confirmation of sale qualification | □No □Yes □Unclear |
| Most concerned factors (multiple selection) | □Ease to use □Price □Choice □Reliability/Quality □Insurance reimbursement □Privacy security □Other__________ |
| **(f) Telemedicine / Virtual visit**  *Introduction by investigator: Telemedicine and virtual visits refer to an innovative format to provide real-time virtual medical services to patients, which are comparable to the in-person services. Such services are usually based on audio and video synchronizations, and they can be provided through business-to-business (between hospitals/doctors, with doctors’ instruction in both sides) or business-to-consumers (hospitals/doctors direct to patients) models. The contents of telemedicine and virtual visits can include disease diagnosis, therapies, drug prescription, follow-up, and so on.* | |
| Use telemedicine / virtual visit before | □No □Yes |
| (If yes) |  |
| Motivations to use | □Inaccessibility to high-quality service □Reference from doctors □Reference from others □Government project □Other________ |
| Disease (multiple selection) | □General symptom/Chronic disease □Severe illness (surgery/tumour) □Other______ |
| Location of service | □At home □Village clinic/Town health centre □County hospital □Other______ |
| Accompanied by doctor | □No □Yes □Unclear |
| Hospital at remote side | □Province-level hospital □City-level hospital □County-level hospital □Other_____ |
| Doctor at remote side | □General practitioner □Specialist □Other_____ |
| Confirmation of doctor identification | □No □Yes □Unclear |
| Physical examination before or during service | □No □Yes □Unclear |
| Price compared to offline purchase | □Same □Higher □Lower □Unclear |
| Satisfied with the service | □No □Yes □Hard to say |
| Encounter medical dispute | □No □Yes □Unclear |
| (If no) |  |
| Willing to use | □No □Yes |
| (Not willing to use) |  |
| Reason (multiple selection) | □Unable to use □High price □Limited choice □Low reliability/quality □No insurance reimbursement □Privacy security □Not necessary □Other__________ |
| (Willing to use) |  |
| Motivation to use | □Inaccessibility to high-quality service □Reference from doctors □Reference from others □Government project □Other________ |
| Disease preferred (multiple selection) | □General symptom/Chronic disease □Severe illness (surgery/tumour) □Other______ |
| Location of service preferred | □At home □Village clinic/Town health centre □County hospital □Other______ □Any/Not specified |
| Accompanied by doctor | □No □Yes □Unclear/Not specified |
| Hospital preferred at remote side | □Province-level hospital □City-level hospital □County-level hospital □Other_____ □Any/Not specified |
| Doctor preferred at remote side | □General practitioner □Specialist □Other_____ |
| Requirement to confirm doctor identification | □No □Yes □Unclear/Not specified |
| Price preferred compared to offline purchase | □Same □Higher □Lower □Unclear/Not specified |
| Most concerned factors (multiple selection) | □Ease to use □Price □Choice □Reliability/Quality □Insurance reimbursement □Privacy security □Other__________ |
| Assumed the price of one face-to-face visit of specialists of tertiary care is CNY1000, how much are you willing to pay for a virtual visit? | □<CNY800 □CNY800-1000 □CNY1000-1200 □CNY1200-1500 □>CNY1500 |

Part II. Doctors survey

| **(a) Demographics** | |
| --- | --- |
| Gender | □Male □Female |
| Age | ____years |
| Education level | □Junior high school or equivalent □Senior high school or equivalent □Junior college □Bachelor degree □Master degree and higher |
| Working facility | □County-level hospital □Town health centre □Village clinic |
| Specialty | □Internal medicine □Surgery □Gynaecology / Obstetrics □Paediatric □Traditional Chinese Medicine □Ear-Nose-Throat □Stomatology □Ophthalmology □Skin □Anaesthesiology □Rehabilitation □Preventive care □Emergency □Others |
| Working year | ____years |
| Professional rank | □Junior □Medium □Senior |
| Actual monthly income (1CNY=0.16USD) | □<CNY1,000 □CNY1,000-1,999 □CNY2,000-5,999 □CNY6,000-9,999 □CNY10,000-19,999 □≥CNY20,000 |
| Expected monthly income  (1CNY=0.16USD) | □<CNY1,000 □CNY1,000-1,999 □CNY2,000-5,999 □CNY6,000-9,999 □CNY10,000-19,999 □≥CNY20,000 |
| **(b) Working environment and self-rated health** | |
| Enough time for patient communication | □Very disagree □Disagree □Neutral □Agree □Very Agree |
| Information and telecommunication technology promote my work | □Very disagree □Disagree □Neutral □Agree □Very Agree |
| Satisfied with current income system | □Very disagree □Disagree □Neutral □Agree □Very Agree |
| Feel too much workload for me | □Very disagree □Disagree □Neutral □Agree □Very Agree |
| Feel being respected by the society | □Very disagree □Disagree □Neutral □Agree □Very Agree |
| Have enough time for sleep | □Very disagree □Disagree □Neutral □Agree □Very Agree |
| Have enough time for exercise | □Very disagree □Disagree □Neutral □Agree □Very Agree |
| Feel well with my physical health | □Very disagree □Disagree □Neutral □Agree □Very Agree |
| Feel well with my mental health | □Very disagree □Disagree □Neutral □Agree □Very Agree |
| **(c) Usage of digital device and internet** | |
| Owning a smartphone | □No □Yes |
| Phone access to internet | □No □Yes |
| Able to use video-chat | □No □Yes |
| Owning an office computer | □No □Yes |
| Computer access to internet | □No □Yes |
| Computer equipped with camera | □No □Yes |
| **(d) E-consultation** | |
| Provide e-consultation before | □No □Yes |
| (If yes) |  |
| Motivations to provide (multiple selection) | □Work needs □Patient request □Improve reputation □Improve income □Other_____ |
| Platform to provide (multiple selection) | □Phone call □Message □WeChat □Official website □Third-party platform □Other_____ |
| Disease to consult about (multiple selection) | □General symptom/Chronic disease □Severe illness (surgery/tumour) □Other______ |
| Content to consult about (multiple selection) | □Diagnosis □Treatment □Fee □Medication use □Follow-up □Other______ |
| Provision of clinical diagnosis | □No □Yes □Unclear |
| Provision of clinical treatment or prescription | □No □Yes □Unclear |
| Confirmation of patient identification | □No □Yes □Unclear |
| Reward from service | CNY______ |
| Expected reward from service | CNY______ |
| Price of service | CNY_____ |
| (If no) |  |
| Willing to adopt ^*^ | □No □Yes |
| (Not willing to adopt) |  |
| Reason (multiple selection) | □Unable to use □Unsafety □Uselessness/Low quality □No income incentive □Other__________ |
| (Willing to use) |  |
| Motivation to adopt | □Work needs □Patient request □Improve reputation □Improve income □Other_____ |
| Platform preferred to provide service | □Phone call □Message □WeChat □Official website □Third-party platform □Other_____ |
| Disease preferred to consult about (multiple selection) | □General symptom/Chronic disease □Severe illness (surgery/tumour) □Other______ |
| Content preferred to consult about (multiple selection) | □Diagnosis □Treatment □Fee □Medication use □Follow-up □Other______ |
| Requirement to confirm patient identification | □No □Yes □Unclear |
| Expected reward from service | CNY______ |
| Most concerned factors (multiple selection) | □Ease to use / Technology preparedness □Income incentive □Policy support □Reliability/Quality □Insurance reimbursement □Privacy security □Medical dispute □Other__________ |
| **(e) Telemedicine / Virtual visit** | |
| Provide telemedicine / virtual visit before | □No □Yes |
| (If yes) |  |
| Motivations to provide (multiple selection) | □Work needs □Request from other hospital/doctor □Patient request □Other_____ |
| Disease (multiple selection) | □General symptom/Chronic disease □Severe illness (surgery/tumour) □Other______ |
| Role played in telemedicine | □Instructor □Being instructed □Other______ |
| Doctor giving instruction | □Province-level hospital □City-level hospital □County-level hospital □Town health centre □Other_____ |
| Doctor receiving instruction | □Same-level hospital □Lower-level hospital □Other______ |
| Doctor at remote side | □General practitioner □Specialist □Other_____ |
| Responsible for medical liability | □No □Yes □Unclear □Other_____ |
| Sufficient technical support | □No □Yes □Unclear |
| Performance of service compared to traditional mode | □Same as normal □Better □Worse □Hard to say |
| Encounter medical dispute | □No □Yes □Unclear |
| **(If no)** |  |
| Willing to adopt ^*^ | □No □Yes |
| (Not willing to adopt) |  |
| Reason (multiple selection) | □Unable to use □Technology preparedness □Unsafety □Uselessness/Low quality □No income incentive □Other__________ |
| (Willing to use) |  |
| Motivation to use (multiple selection) | □Work needs □Request from other hospital/doctor □Patient request □Other_____ |
| Disease preferred (multiple selection) | □General symptom/Chronic disease □Severe illness (surgery/tumour) □Other______ |
| Requirement of doctor at patient side | □No □Yes □Unclear/Not specified |
| Requirement of patient’s medical history | □No □Yes □Unclear/Not specified |
| Requirement of patient’s consent | □No □Yes □Unclear/Not specified |
| Requirement to confirm patient identification | □No □Yes □Unclear/Not specified |
| Requirement to contact patient face-to-face before service | □No □Yes □Unclear/Not specified |
| Requirement to perform physical examination for patient | □No □Yes □Unclear/Not specified |
| Requirement of regulation and standard specific for telemedicine | □No □Yes □Unclear/Not specified |
| Expected rewards from telemedicine | □Same as offline mode □Higher □Lower □Unclear/Not specified |
| Perceived performance of telemedicine compared to normal care | □Same as normal □Better □Worse □Hard to say |
| Perceived advantages of telemedicine (multiple selection) | □Increase income □Improve reputation □Improve medical performance □Increase health accessibility □Improve medical skills □Other__________ |
| Perceived disadvantages of telemedicine (multiple selection) | □Technology preparedness □Training □Instruction from higher-level doctors □Medical safety/quality □Income incentive □Policy support □Insurance reimbursement □Medical dispute □Other__________ |

* Questions regarding the willingness to adopt online consultation and telemedicine were only asked for county-level doctors.
