# Supplementary material for: A profile of patients’ and doctors’ perceptions, acceptance, and utilization of e-health in a deprived region in southwestern China
Source: PLOS Digit Health. 2023 Apr 25;2(4):e0000238. doi: 10.1371/journal.pdig.0000238 (PMC10129013; doi:10.1371/journal.pdig.0000238)
Supplement: S2 Appendix — (DOCX) [file pdig.0000238.s002.docx]

# S2 Appendix. Patients’ responses to survey items by types of service and user groups

| Question & Item | Total sample | Use before | Willing to use | Reluctant to use |  |
| --- | --- | --- | --- | --- | --- |
|  |  | Number (%) | Number (%) | Number (%) |  |
| **1. Any e-health service** | **N=485** | **145 (29.9)** | **148 (30.5)** | **192 (39.6)** |  |
| **2. Medical appointment** | **N=485** | **60 (12.4)** | **147 (30.3)** | **278 (57.3)** |  |
| 2.1 Concerned factors (multiple responses) |  |  |  |  |  |
| Ease to use |  | 1 (1.7) | 4 (2.7) | 188 (67.6) |  |
| Price |  | 12 (20.0) | 38 (25.9) | 9 (3.2) |  |
| Choice |  | 8 (13.3) | 15 (10.2) | 13 (4.7) |  |
| Reliability/Quality |  | 42 (70.0) | 102 (69.4) | 55 (19.8) |  |
| Reimbursement |  | 15 (25.0) | 28 (19.0) | 15 (5.4) |  |
| Privacy |  | 9 (15.0) | 29 (19.7) | 15 (5.4) |  |
| Other (Not necessity) |  | 0 (0.0) | 0 (0.0) | 26 (9.4) |  |
| Missing data |  | 0 (0.0) | 0 (0.0) | 0 (0) |  |
| 2.2 Manner of appointment |  |  |  |  |  |
| Official website / hotline |  | 37 (63.8) | 87 (84.5) |  |  |
| Third-party platform |  | 13 (22.4) | 16 (15.5) |  |  |
| Others |  | 8 (13.8) | 0 (0.0) |  |  |
| Missing data |  | 2 (3.3) | 44 (29.9) |  |  |
| 2.3 Level of facility |  |  |  |  |  |
| Province/City-level |  | 31 (54.4) | 40 (27.6) |  |  |
| County-level |  | 19 (33.3) | 52 (10.7) |  |  |
| Health centre |  | 1 (1.8) | 0 (0.0) |  |  |
| Any/Not specified |  | 6 (10.5) | 53 (61.7) |  |  |
| Missing data |  | 3 (5.0) | 2 (1.4) |  |  |
| 2.4 Level of doctor |  |  |  |  |  |
| General practitioner |  | 14 (23.7) | 20 (13.7) |  |  |
| Specialist |  | 30 (50.8) | 97 (66.4) |  |  |
| Any/Not specified |  | 15 (25.4) | 29 (19.9) |  |  |
| Missing data |  | 1 (1.7) | 1 (0.7) |  |  |
| 2.5 Charge compared to normal |  |  |  |  |  |
| Same |  | 26 (45.6) | 48 (38.7) |  |  |
| Lower |  | 7 (12.3) | 30 (24.2) |  |  |
| Higher |  | 4 (7.0) | 7 (5.6) |  |  |
| Not specified |  | 20 (35.1) | 39 (31.5) |  |  |
| Missing data |  | 3 (5.0) | 23 (15.6) |  |  |
| 2.6 Satisfaction |  |  |  |  |  |
| Yes |  | 40 (81.6) |  |  |  |
| No |  | 3 (6.1) |  |  |  |
| Not specified |  | 6 (12.2) |  |  |  |
| Missing data |  | 11 (18.3) |  |  |  |
| **3. Online consultation** | **N=475** | **87 (18.3)** | **132 (27.8)** | **256 (53.9)** |  |
| 3.1 Concerned factors (multiple responses) |  |  |  |  |  |
| Ease to use |  | 34 (39.1) | 48 (36.4) | 174 (68.0) |  |
| Price |  | 21 (24.1) | 43 (32.6) | 17 (6.6) |  |
| Choice |  | 11 (12.6) | 18 (13.6) | 15 (5.9) |  |
| Reliability/quality |  | 51 (58.6) | 83 (62.9) | 32 (12.5) |  |
| Reimbursement |  | 16 (18.4) | 20 (15.2) | 25 (9.8) |  |
| Privacy |  | 15 (17.2) | 26 (19.7) | 23 (9.0) |  |
| Other (Not necessity) |  | 0 (0.0) | 2 (1.5) | 6 (2.3) |  |
| Missing data |  | 0 (0.0) | 0 (0) | 0 (0) |  |
| 3.2 Motivation to use |  |  |  |  |  |
| Inaccessibility to service |  | 13 (14.9) | 54 (40.9) |  |  |
| Inconvenience to service |  | 38 (43.7) | 58 (43.9) |  |  |
| Reference from others |  | 20 (23.0) | 19 (14.4) |  |  |
| Not specified/others (follow-up) |  | 16 (18.4) | 1 (0.8) |  |  |
| Missing data |  | 0 (0.0) | 0 (0) |  |  |
| 3.3 Level of facility |  |  |  |  |  |
| Province/City-level |  | 35 (42.7) | 45 (36.3) |  |  |
| County-level |  | 23 (28.0) | 44 (35.5) |  |  |
| Health centre |  | 0 (0.0) | 3 (2.4) |  |  |
| Any/Not specified |  | 23 (28.1) | 32 (25.8) |  |  |
| Missing data |  | 5 (5.7) | 8 (6.1) |  |  |
| 3.4 Level of doctor |  |  |  |  |  |
| General practitioner |  | 17 (20.5) | 13 (10.5) |  |  |
| Specialist |  | 50 (60.2) | 81 (65.3) |  |  |
| Any/Not specified |  | 16 (19.2) | 30 (24.1) |  |  |
| Missing data |  | 4 (4.6) | 8 (6.1) |  |  |
| 3.5 Confirmation of doctor |  |  |  |  |  |
| Yes |  | 37 (45.7) | 85 (66.9) |  |  |
| No |  | 19 (23.5) | 19 (15.0) |  |  |
| Not specified/unclear |  | 25 (30.9) | 23 (18.1) |  |  |
| Missing data |  | 6 (6.9) | 5 (3.8) |  |  |
| 3.6 Disease |  |  |  |  |  |
| Chronic disease / General symptoms |  | 45 (57.7) | 44 (36.4) |  |  |
| Severe disease (surgery / tumour) |  | 25 (32.1) | 52 (43.0) |  |  |
| Not specified |  | 8 (10.3) | 25 (20.7) |  |  |
| Missing data |  | 9 (10.3) | 11 (8.3) |  |  |
| 3.7 Content (multiple responses) |  |  |  |  |  |
| Diagnosis |  | 56 (71.8) | 90 (68.2) |  |  |
| Treatment |  | 38 (48.7) | 77 (58.3) |  |  |
| Fee |  | 15 (19.2) | 35 (26.5) |  |  |
| Medication |  | 21 (26.9) | 42 (31.8) |  |  |
| Follow-up |  | 10 (12.8) | 24 (18.2) |  |  |
| Others |  | 2 (2.6) | 2 (1.5) |  |  |
| Missing data |  | 0 (0.0) | 0 (0.0) |  |  |
| 3.8 Charge |  |  |  |  |  |
| Free |  | 41 (77.4) | 28 (43.8) |  |  |
| CNY1-30 (US$1-5) |  | 10 (18.9) | 29 (45.3) |  |  |
| >CNY30 (>US$5) |  | 2 (3.8) | 7 (10.9) |  |  |
| Missing data |  | 25 (28.7) | 68 (51.5) |  |  |
| 3.9 Satisfaction |  |  |  |  |  |
| Yes |  | 57 (89.1) |  |  |  |
| No |  | 2 (3.1) |  |  |  |
| Not specified |  | 5 (7.8) |  |  |  |
| Missing data |  | 23 (26.4) |  |  |  |
| **4. Drug purchase** | **N=474** | **37 (7.8)** | **66 (13.9)** | **371 (78.3)** |  |
| 4.1 Concerned factors (multiple responses) |  |  |  |  |  |
| Ease to use |  | 12 (32.4) | 27 (40.9) | 197 (53.1) |  |
| Price |  | 10 (27.0) | 18 (27.3) | 30 (8.1) |  |
| Variety |  | 4 (10.8) | 20 (30.3) | 18 (4.9) |  |
| Reliability/quality |  | 25 (67.6) | 45 (68.2) | 159 (42.9) |  |
| Reimbursement |  | 3 (8.1) | 12 (18.2) | 34 (9.2) |  |
| Privacy |  | 7 (18.9) | 12 (18.2) | 19 (5.1) |  |
| Other (Not necessity) |  | 0 (0.0) | 0 (0.0) | 12 (3.2) |  |
| Missing data |  | 0 (0) | 0 (0) | 0 (0) |  |
| 4.2 Motivation to use |  |  |  |  |  |
| Inaccessibility to drug |  | 10 (30.3) | 19 (28.8) |  |  |
| Saving time |  | 7 (21.2) | 27 (40.9) |  |  |
| Reference from others |  | 14 (42.4) | 14 (21.2) |  |  |
| Not specified/others (follow-up, saving money) |  | 2 (6.1) | 5 (7.6) |  |  |
| Missing data |  | 4 (10.8) | 1 (1.5) |  |  |
| 4.3 Charge compared to normal |  |  |  |  |  |
| Same |  | 9 (25.7) | 12 (27.3) |  |  |
| Lower |  | 15 (42.9) | 24 (54.5) |  |  |
| Higher |  | 4 (11.4) | 1 (2.3) |  |  |
| Not specified |  | 7 (20.0) | 7 (15.9) |  |  |
| Missing data |  | 2 (5.4) | 22 (33.3) |  |  |
| 4.4 Confirmation of sale qualification |  |  |  |  |  |
| Yes |  | 15 (42.9) | 38 (76.0) |  |  |
| No |  | 10 (28.6) | 4 (8.0) |  |  |
| Not specified/unclear |  | 10 (28.6) | 8 (16.0) |  |  |
| Missing data |  | 2 (5.4) | 16 (24.2) |  |  |
| 4.5 Type of drug |  |  |  |  |  |
| Rx drug |  | 4 (12.9) |  |  |  |
| Generic drug |  | 16 (51.6) |  |  |  |
| Not specified |  | 11 (35.5) |  |  |  |
| Missing data |  | 6 (16.2) |  |  |  |
| 4.6 Requirement on doctor prescription |  |  |  |  |  |
| Yes |  | 0 (0.0) |  |  |  |
| No |  | 10 (76.9) |  |  |  |
| Not specified/unclear |  | 3 (23.1) |  |  |  |
| Missing data |  | 24 (64.9) |  |  |  |
| 4.7 Satisfaction |  |  |  |  |  |
| Yes |  | 26 (83.9) |  |  |  |
| No |  | 3 (9.7) |  |  |  |
| Not specified |  | 2 (6.5) |  |  |  |
| Missing data |  | 6 (16.2) |  |  |  |
| **5. Telemedicine / Telehealth** | **N=468** | **28 (6.0)** | **135 (28.8)** | **305 (62.9)** |  |
| 5.1 Concerned factors (multiple responses) |  |  |  |  |  |
| Ease to use |  | 7 (25.0) | 50 (37.0) | 207 (67.9) |  |
| Price |  | 9 (32.1) | 48 (35.6) | 29 (9.5) |  |
| Choice |  | 5 (17.9) | 25 (18.5) | 19 (6.2) |  |
| Reliability/quality |  | 20 (71.4) | 88 (65.2) | 63 (20.7) |  |
| Reimbursement |  | 4 (14.3) | 24 (17.8) | 19 (6.2) |  |
| Privacy |  | 5 (17.9) | 18 (13.3) | 14 (4.6) |  |
| Other (Not necessity, Hard to hospitalization) |  | 1 (3.6) | 3 (2.2) | 27 (8.9) |  |
| Missing data |  | 0 (0) | 0 (0) | 0 (0) |  |
| 5.2 Motivation to use |  |  |  |  |  |
| Inaccessibility to high-quality service |  | 3 (10.7) | 67 (49.6) |  |  |
| Reference from doctors |  | 16 (57.1) | 40 (29.6) |  |  |
| Government promotional campaign |  | 2 (7.1) | 5 (3.7) |  |  |
| Reference from others |  | 5 (17.9) | 6 (4.4) |  |  |
| Not specified/others (saving time) |  | 2 (7.1) | 17 (12.6) |  |  |
| Missing data |  | 0 (0) | 0 (0) |  |  |
| 5.3 Disease |  |  |  |  |  |
| Chronic disease / General symptoms |  | 10 (35.7) | 25 (20.0) |  |  |
| Severe disease (surgery / tumour) |  | 15 (53.6) | 87 (69.6) |  |  |
| Not specified |  | 3 (10.7) | 13 (10.4) |  |  |
| Missing data |  | 0 (0.0) | 10 (7.4) |  |  |
| 5.4 Location of patient |  |  |  |  |  |
| Home |  | 9 (34.6) | 26 (20.5) |  |  |
| Health centre/ Village clinic |  | 5 (19.2) | 37 (29.1) |  |  |
| County-level hospital |  | 8 (30.8) | 29 (22.8) |  |  |
| Any/others |  | 4 (15.4) | 35 (27.6) |  |  |
| Missing data |  | 2 (7.1) | 8 (5.9) |  |  |
| 5.5 Accompanied by doctors at patients’ side |  |  |  |  |  |
| Yes |  | 17 (63.0) | 74 (56.9) |  |  |
| No |  | 6 (22.2) | 16 (12.3) |  |  |
| Unclear |  | 4 (14.8) | 40 (30.8) |  |  |
| Missing data |  | 1 (3.6) | 5 (3.7) |  |  |
| 5.6 Level of hospital at remote site |  |  |  |  |  |
| Province/City-level |  | 20 (71.4) | 86 (66.2) |  |  |
| County-level |  | 6 (21.4) | 24 (18.5) |  |  |
| Health centre |  | 0 (0.0) | 2 (1.5) |  |  |
| Any/Not specified |  | 2 (7.2) | 18 (13.8) |  |  |
| Missing data |  | 0 (0.0) | 5 (3.7) |  |  |
| 5.7 Level of doctor |  |  |  |  |  |
| General practitioner |  | 3 (10.7) | 14 (10.9) |  |  |
| Specialist |  | 20 (71.4) | 93 (72.1) |  |  |
| Any/Not specified |  | 5 (17.9) | 22 (17.1) |  |  |
| Missing data |  | 0 (0.0) | 6 (4.4) |  |  |
| 5.8 Confirmation of doctor |  |  |  |  |  |
| Yes |  | 10 (37.0) | 87 (68.5) |  |  |
| No |  | 7 (25.9) | 21 (16.5) |  |  |
| Not specified/unclear |  | 10 (37.0) | 19 (15.0) |  |  |
| Missing data |  | 1 (3.6) | 8 (5.9) |  |  |
| 5.9 Charge |  |  |  |  |  |
| Same |  | 2 (14.3) | 28 (29.5) |  |  |
| Lower |  | 0 (0.0) | 18 (18.9) |  |  |
| Higher |  | 4 (28.6) | 22 (23.2) |  |  |
| Not specified |  | 8 (57.1) | 27 (28.4) |  |  |
| Missing data |  | 14 (50.0) | 40 (29.6) |  |  |
| 5.10 Complete physical examination |  |  |  |  |  |
| Yes |  | 12 (52.2) |  |  |  |
| No |  | 6 (26.1) |  |  |  |
| Not specified/unclear |  | 5 (21.7) |  |  |  |
| Missing data |  | 5 (17.9) |  |  |  |
| 5.11 Satisfaction |  |  |  |  |  |
| Yes |  | 11 (44.0) |  |  |  |
| No |  | 1 (4.0) |  |  |  |
| Not specified |  | 13 (52.0) |  |  |  |
| Missing data |  | 3 (10.7) |  |  |  |
| 5.12 Encounter medical dispute |  |  |  |  |  |
| Yes |  | 12 (48.0) |  |  |  |
| No |  | 8 (32.0) |  |  |  |
| Not specified |  | 5 (20.0) |  |  |  |
| Missing data |  | 3 (10.7) |  |  |  |
| 5.13 Willingness to pay compared to face-to-face visit (CNY1,000 or US$160) |  |  |  |  |  |
| ≤CNY800 (≤US$128) |  | 10 (38.5) | 57 (51.4) |  |  |
| CNY801-1000 (US$128-160) |  | 10 (38.5) | 40 (36.0) |  |  |
| CNY1001-1200 (US$160-192) |  | 4 (15.4) | 12 (10.8) |  |  |
| CNY1201-1500 (US$192-240) |  | 1 (3.8) | 1 (0.9) |  |  |
| >CNY1500 (>US$240) |  | 1 (3.8) | 1 (0.9) |  |  |
| Missing data |  | 2 (7.1) | 24 (17.8) |  |  |
