# Supplementary material for: A profile of patients’ and doctors’ perceptions, acceptance, and utilization of e-health in a deprived region in southwestern China
Source: PLOS Digit Health. 2023 Apr 25;2(4):e0000238. doi: 10.1371/journal.pdig.0000238 (PMC10129013; doi:10.1371/journal.pdig.0000238)
Supplement: S3 Appendix — (DOCX) [file pdig.0000238.s003.docx]

# S3 Appendix. Univariate and multivariable analyses of factors associated with patients’ utilization and intention to adopt e-health service

| **Medical Appointment** | **Utilization (n=485)** | | | | **Willing to use (n=485)** | | | |
| --- | --- | --- | --- | --- | --- | --- | --- | --- |
|  | **Yes (%)** | **χ² test** | **Crude OR** | **Adjusted OR^a^** | **Yes (%) ^b^** | **χ² test** | **Crude OR** | **Adjusted OR^a^** |
| **Sex** |  | 1.693 (P=0.193) |  |  |  | 1.205 (P=0.272) |  |  |
| Male | 17 (9.8) |  | Ref | Ref | 80 (46.0) |  | Ref | Ref |
| Female | 43 (13.8) |  | 1.474 (0.813-2.672) | 1.489 (0.767-2.886) (P=0.240) | 127 (40.8) |  | 0.809 (0.557-1.176) | 0.809 (0.512-1.279) (P=0.364) |
| **Age group** |  | 6.615 (P=0.085) |  |  |  | 27.907 (P=0.000) |  |  |
| 16-30 | 32 (16.1) |  | Ref | Ref | 111 (55.8) |  | Ref | Ref |
| 31-45 | 16 (11.3) |  | 0.664 (0.349-1.263) | 0.780 (0.351-1.732) (P=0.542) | 54 (38.0) |  | 0.485 (0.313-0.753) | 0.685 (0.388-1.207) (P=0.190) |
| 46-60 | 6 (5.9) |  | 0.327 (0.132-0.810) | 1.055 (0.392-2.838) (P=0.916) | 26 (25.7) |  | 0.274 (0.162-0.464) | 1.150 (0.576-2.296) (P=0.691) |
| >60 ^c^ | 5 (11.9) |  | 0.704 (0.257-1.928) | (Combined with 46-60 group) | 15 (35.7) |  | 0.440 (0.220-0.877) | (Combined with 46-60 group) |
| **Education** |  | 17.068 (P=0.001) |  |  |  | 58.609 (P=0.000) |  |  |
| Primary and lower | 12 (7.0) |  | Ref | Ref | 45 (26.2) |  | Ref | Ref |
| Junior high school | 17 (12.1) |  | 1.829 (0.842-3.971) | 0.866 (0.330-2.268) (P=0.769) | 57 (40.4) |  | 1.909 (1.184-3.080) | 1.085 (0.587-2.010) (P=0.793) |
| Senior high school | 12 (12.6) |  | 1.915 (0.824-4.450) | 0.584 (0.182-1.879) (P=0.367) | 47 (49.5) |  | 2.761 (1.631-4.675) | 1.089 (0.508-2.335) (P=0.827) |
| College and higher | 19 (26.0) |  | 4.668 (2.127-10.242) | 1.143 (0.300-4.354) (P=0.844) | 57 (78.1) |  | 10.045 (5.242-19.251) | 3.281 (1.170-9.207) (P=0.024) |
| **Occupation** |  | 12.654 (P=0.005) |  |  |  | 44.169 (P=0.000) |  |  |
| Farmer | 18 (7.3) |  | Ref | Ref | 73 (29.7) |  | Ref | Ref |
| Public or private sector | 11 (20.0) |  | 3.175 (1.403-7.184) | 0.828 (0.224-3.056) (P=0.777) | 39 (70.9) |  | 5.767 (3.032-10.970) | 1.627 (0.622-4.259) (P=0.321) |
| Freelancer and other work | 15 (16.5) |  | 2.509 (1.206-5.221) | 1.640 (0.685-3.927) (P=0.267) | 41 (45.1) |  | 1.944 (1.185-3.191) | 1.039 (0.561-1.925) (P=0.903) |
| Student, unemployed, retired | 16 (18.0) |  | 2.788 (1.353-5.745) | 1.483 (0.546-4.027) (P=0.439) | 52 (58.4) |  | 3.323 (2.010-5.492) | 1.381 (0.687-2.776) (P=0.364) |
| **Work outside** |  | 5.585 (P=0.018) |  |  |  | 15.546 (P=0.000) |  |  |
| No | 45 (10.9) |  | Ref | Ref | 161 (39.0) |  | Ref | Ref |
| Yes | 15 (20.8) |  | 2.147 (1.123-4.102) | 2.323 (1.114-4.840) (P=0.024) | 46 (63.9) |  | 2.769 (1.646-4.657) | 2.010 (1.105-3.655) (P=0.022) |
| **Residence** |  | 16.215 (P=0.000) |  |  |  | 22.425 (P=0.000) |  |  |
| Rural | 37 (9.4) |  | Ref | Ref | 147 (37.5) |  | Ref | Ref |
| Township | 23 (24.7) |  | 3.162 (1.770-5.647) | 1.276 (0.543-2.998) (P=0.576) | 60 (64.5) |  | 3.028 (1.890-4.852) | 1.073 (0.538-2.136) (P=0.842) |
| **Cohabitant** |  | 5.765 (P=0.056) |  |  |  | 6.419 (P=0.040) |  |  |
| <=3 | 16 (16.2) |  | Ref | Ref | 49 (49.5) |  | Ref | Ref |
| 4to5 | 35 (13.9) |  | 0.835 (0.439-1.589) | 1.055 (0.502-2.221) (P=0.887) | 109 (43.3) |  | 0.779 (0.489-1.242) | 1.105 (0.629-1.941) (P=0.727) |
| >6 | 7 (6.2) |  | 0.342 (0.134-0.870) | 0.477 (0.168-1.354) (P=0.164) | 37 (32.7) |  | 0.496 (0.284-0.865) | 0.914 (0.467-1.788) (P=0.792) |
| **Household income** (1CNY=0.16USD) |  | 11.368 (P=0.010) |  |  |  | 25.073 (P=0.000) |  |  |
| <CNY20000 | 17 (9.3) |  | Ref | Ref | 54 (29.7) |  | Ref | Ref |
| CNY20001-30000 | 9 (9.3) |  | 1.000 (0.428-2.336) | 0.782 (0.314-1.946) (P=0.597) | 44 (45.4) |  | 1.968 (1.181-3.280) | 1.677 (0.927-3.031) (P=0.087) |
| CNY30001-50000 | 8 (9.8) |  | 1.060 (0.438-2.565) | 0.708 (0.261-1.916) (P=0.496) | 37 (45.1) |  | 1.944 (1.134-3.333) | 1.388 (0.738-2.612) (P=0.309) |
| >CNY50000 | 26 (21.0) |  | 2.592 (1.339-5.018) | 1.380 (0.599-3.177) (P=0.449) | 72 (58.1) |  | 3.282 (2.035-5.293) | 1.379 (0.743-2.557) (P=0.308) |
| **Outpatient service** |  | 3.321 (P=0.068) |  |  |  | 2.030 (P=0.154) |  |  |
| No | 41 (10.9) |  | Ref | Ref | 154 (41.0) |  | Ref | Ref |
| Yes | 19 (17.4) |  | 1.722 (0.953-3.111) | 1.392 (0.690-2.809) (P=0.355) | 53 (48.6) |  | 1.361 (0.887-2.088) | 1.127 (0.655-1.943) (P=0.665) |
| **Inpatient service** |  | 1.856 (P=0.173) |  |  |  | 0.270 (P=0.603) |  |  |
| No | 40 (14.1) |  | Ref | Ref | 124 (43.7) |  | Ref | Ref |
| Yes | 20 (10.0) |  | 0.677 (0.383-1.197) | 1.113 (0.555-2.232) (P=0.762) | 83 (41.3) |  | 0.906 (0.629-1.307) | 1.241 (0.768-2.006) (P=0.377) |
| **Smartphone** |  | 11.593 (P=0.003) |  |  |  | 62.387 (P=0.000) |  |  |
| Not own | 8 (5.6) |  | Ref | Ref | 27 (18.9) |  | Ref | Ref |
| Own, without network | 6 (9.0) |  | 1.667 (0.554-5.013) | 1.188 (0.360-3.920) (P=0.778) | 21 (31.3) |  | 1.955 (1.006-3.800) | 2.284 (1.089-4.792) (P=0.029) |
| Own, with network | 46 (16.7) |  | 3.380 (1.549-7.375) | 1.374 (0.515-3.669) (P=0.525) | 159 (57.8) |  | 5.877 (3.628-9.521) | 3.979 (2.081-7.599) (P=0.000) |
| **Personal computer** |  | 25.498 (P=0.000) |  |  |  | 38.970 (P=0.000) |  |  |
| Not own | 18 (6.2) |  | Ref | Ref | 91 (31.4) |  | Ref | Ref |
| Own, without network | 9 (19.6) |  | 3.688 (1.544-8.810) | 2.835 (1.059-7.599) (P=0.038) | 24 (52.2) |  | 2.386 (1.271-4.477) | 1.323 (0.631-2.776) (P=0.458) |
| Own, with network | 33 (22.1) |  | 4.292 (2.323-7.931) | 2.757 (1.215-6.246) (P=0.015) | 92 (61.7) |  | 3.519 (2.328-5.320) | 1.473 (0.838-2.588) (P=0.178) |
| **Online consultation** | **Utilization (n=475)** | | | | **Willing to use (n=475)** | | | |
|  | **Yes (%)** | **χ² test** | **Crude OR** | **Adjusted OR^a^** | **Yes (%) ^b^** | **χ² test** | **Crude OR** | **Adjusted OR^a^** |
| **Sex** |  | 0.089 (P=0.765) |  |  |  | 0.070 (P=0.791) |  |  |
| Male | 30 (17.2) |  | Ref | Ref | 77 (45.3) |  | Ref | Ref |
| Female | 57 (18.3) |  | 1.078 (0.663-1.755) | 1.342 (0.768-2.344) (P=0.301) | 142 (46.6) |  | 1.054 (0.723-1.536) | 1.332 (0.837-2.121) (P=0.227) |
| **Age group** |  | 4.247 (P=0.236) |  |  |  | 25.467 (P=0.000) |  |  |
| 16-30 | 43 (21.6) |  | Ref | Ref | 116 (59.2) |  | Ref | Ref |
| 31-45 | 20 (14.1) |  | 0.596 (0.333-1.065) | 0.969 (0.480-1.958) (P=0.931) | 55 (39.3) |  | 0.446 (0.287-0.695) | 0.729 (0.412-1.290) (P=0.279) |
| 46-60 | 15 (14.9) |  | 0.636 (0.334-1.210) | 2.048 (0.888-4.721) (P=0.093) | 30 (30.9) |  | 0.308 (0.184-0.516) | 1.332 (0.664-2.672) (P=0.419) |
| >60 ^c^ | 9 (21.4) |  | 0.988 (0.439-2.223) | (Combined with 46-60 group) | 17 (41.5) |  | 0.489 (0.247-0.968) | (Combined with 46-60 group) |
| **Education** |  | 24.236 (P=0.000) |  |  |  | 52.911 (P=0.000) |  |  |
| Primary and lower | 18 (10.5) |  | Ref | Ref | 47 (27.8) |  | Ref | Ref |
| Junior high school | 19 (13.5) |  | 1.330 (0.669-2.644) | 1.250 (0.537-2.912) (P=0.605) | 59 (43.7) |  | 2.016 (1.249-3.253) | 1.029 (0.549-1.927) (P=0.929) |
| Senior high school | 28 (29.5) |  | 3.567 (1.847-6.887) | 2.438 (0.939-6.328) (P=0.067) | 58 (61.7) |  | 4.184 (2.450-7.143) | 1.332 (0.614-2.886) (P=0.467) |
| College and higher | 22 (30.1) |  | 3.670 (1.825-7.383) | 2.570 (0.794-8.331) (P=0.115) | 53 (72.6) |  | 6.881 (3.722-12.724) | 1.998 (0.706-5.646) (P=0.192) |
| **Occupation** |  | 16.131 (P=0.001) |  |  |  | 44.746 (P=0.000) |  |  |
| Farmer | 28 (11.4) |  | Ref | Ref | 77 (31.8) |  | Ref | Ref |
| Public or private sector | 16 (29.1) |  | 3.190 (1.580-6.439) | 1.876 (0.640-5.501) (P=0.252) | 37 (68.5) |  | 4.664 (2.472-8.799) | 2.052 (0.763-5.518) (P=0.154) |
| Freelancer and other work | 21 (23.1) |  | 2.335 (1.248-4.369) | 1.840 (0.860-3.943) (P=0.116) | 46 (52.9) |  | 2.409 (1.460-3.973) | 1.514 (0.813-2.824) (P=0.191) |
| Student, unemployed, retired | 22 (24.7) |  | 2.549 (1.369-4.748) | 1.706 (0.736-3.955) (P=0.213) | 57 (64.8) |  | 3.948 (2.361-6.603) | 1.994 (0.976-4.071) (P=0.058) |
| **Work outside** |  | 4.102 (P=0.043) |  |  |  | 16.676 (P=0.000) |  |  |
| No | 68 (16.5) |  | Ref | Ref | 171 (42.2) |  | Ref | Ref |
| Yes | 19 (26.4) |  | 1.815 (1.011-3.259) | 1.559 (0.794-3.062) (P=0.197) | 48 (68.6) |  | 2.992 (1.741-5.144) | 2.425 (1.298-4.527) (P=0.005) |
| **Residence** |  | 6.252 (P=0.012) |  |  |  | 14.919 (P=0.000) |  |  |
| Rural | 62 (15.8) |  | Ref | Ref | 160 (41.8) |  | Ref | Ref |
| Township | 25 (26.9) |  | 1.961 (1.151-3.341) | 0.860 (0.399-1.855) (P=0.701) | 59 (64.1) |  | 2.486 (1.551-3.986) | 0.759 (0.376-1.530) (P=0.440) |
| **Cohabitant** |  | 2.413 (P=0.299) |  |  |  | 3.382 (P=0.184) |  |  |
| <=3 | 21 (21.2) |  | Ref | Ref | 45 (47.4) |  | Ref | Ref |
| 4to5 | 46 (18.3) |  | 0.833 (0.467-1.484) | 1.059 (0.554-2.022) (P=0.863) | 118 (47.0) |  | 0.984 (0.613-1.579) | 1.454 (0.814-2.599) (P=0.206) |
| >6 | 15 (13.3) |  | 0.570 (0.276-1.179) | 0.855 (0.380-1.919) (P=0.703) | 40 (37.0) |  | 0.652 (0.372-1.142) | 1.174 (0.589-2.340) (P=0.648) |
| **Household income** (1CNY=0.16USD) |  | 7.805 (P=0.050) |  |  |  | 35.651 (P=0.000) |  |  |
| <CNY20000 | 23 (12.6) |  | Ref | Ref | 55 (30.4) |  | Ref | Ref |
| CNY20001-30000 | 16 (16.5) |  | 1.371 (0.686-2.738) | 1.247 (0.586-2.656) (P=0.567) | 44 (46.8) |  | 2.014 (1.204-3.368) | 1.629 (0.899-2.954) (P=0.107) |
| CNY30001-50000 | 18 (22.0) |  | 1.956 (0.990-3.868) | 1.377 (0.635-2.989) (P=0.418) | 43 (53.8) |  | 2.660 (1.548-4.573) | 1.912 (1.015-3.600) (P=0.045) |
| >CNY50000 | 30 (24.2) |  | 2.215 (1.215-4.036) | 1.172 (0.548-2.504) (P=0.682) | 77 (64.2) |  | 4.106 (2.517-6.698) | 1.939 (1.035-3.636) (P=0.039) |
| **Outpatient service** |  | 1.590 (P=0.207) |  |  |  | 3.194 (P=0.074) |  |  |
| No | 63 (16.8) |  | Ref | Ref | 164 (44.0) |  | Ref | Ref |
| Yes | 24 (22.0) |  | 1.397 (0.824-2.368) | 1.048 (0.558-1.970) (P=0.885) | 55 (53.9) |  | 1.488 (0.959-2.310) | 1.074 (0.614-1.878) (P=0.802) |
| **Inpatient service** |  | 0.051 (P=0.821) |  |  |  | 1.151 (P=0.283) |  |  |
| No | 50 (17.6) |  | Ref | Ref | 133 (48.2) |  | Ref | Ref |
| Yes | 37 (18.4) |  | 1.056 (0.660-1.688) | 1.499 (0.835-2.691) (P=0.175) | 86 (43.2) |  | 0.817 (0.566-1.179) | 1.093 (0.676-1.766) (P=0.716) |
| **Smartphone** |  | 12.344 (P=0.002) |  |  |  | 53.020 (P=0.000) |  |  |
| Not own | 15 (10.5) |  | Ref | Ref | 36 (25.7) |  | Ref | Ref |
| Own, without network | 8 (11.9) |  | 1.151 (0.463-2.866) | 0.992 (0.351-2.801) (P=0.987) | 19 (29.7) |  | 1.221 (0.633-2.355) | 1.116 (0.519-2.404) (P=0.778) |
| Own, with network | 64 (23.3) |  | 2.589 (1.416-4.735) | 1.759 (0.773-4.003) (P=0.178) | 164 (60.5) |  | 4.428 (2.822-6.948) | 2.557 (1.361-4.807) (P=0.004) |
| **Personal computer** |  | 16.893 (P=0.000) |  |  |  | 40.971 (P=0.000) |  |  |
| Not own | 35 (12.1) |  | Ref | Ref | 96 (34.0) |  | Ref | Ref |
| Own, without network | 12 (26.1) |  | 2.566 (1.216-5.415) | 1.418 (0.600-3.350) (P=0.426) | 27 (60.0) |  | 2.912 (1.527-5.551) | 1.649 (0.787-3.459) (P=0.185) |
| Own, with network | 40 (26.8) |  | 2.660 (1.603-4.412) | 1.537 (0.770-3.068) (P=0.223) | 96 (64.9) |  | 3.589 (2.363-5.451) | 1.898 (1.067-3.374) (P=0.029) |
| **Drug purchase** | **Utilization (n=474)** | | | | **Willing to use (n=474)** | | | |
|  | **Yes (%)** | **χ² test** | **Crude OR** | **Adjusted OR^a^** | **Yes (%) ^b^** | **χ² test** | **Crude OR** | **Adjusted OR^a^** |
| **Sex** |  | 0.379 (P=0.538) |  |  |  | 0.505 (P=0.477) |  |  |
| Male | 15 (8.6) |  | Ref | Ref | 40 (23.5) |  | Ref | Ref |
| Female | 22 (7.1) |  | 0.812 (0.410-1.610) | 0.851 (0.376-1.927) (P=0.699) | 63 (20.7) |  | 0.850 (0.542-1.333) | 0.840 (0.491-1.438) (P=0.525) |
| **Age group** |  | 4.572 (P=0.206) |  |  |  | 17.050 (P=0.001) |  |  |
| 16-30 | 19 (9.5) |  | Ref | Ref | 59 (30.1) |  | Ref | Ref |
| 31-45 | 11 (7.7) |  | 0.795 (0.366-1.727) | 1.422 (0.499-4.051) (P=0.510) | 27 (19.4) |  | 0.559 (0.333-0.940) | 0.875 (0.449-1.704) (P=0.694) |
| 46-60 | 7 (6.9) |  | 0.706 (0.287-1.740) | 3.714 (0.946-14.571) (P=0.060) | 12 (12.4) |  | 0.329 (0.167-0.647) | 1.104 (0.476-2.557) (P=0.818) |
| >60 ^c^ | 0 (0.0) |  | N/A | (Combined with 46-60 group) | 4 (9.8) |  | 0.252 (0.086-0.740) | (Combined with 46-60 group) |
| **Education** |  | 32.336 (P=0.000) |  |  |  | 39.940 (P=0.000) |  |  |
| Primary and lower | 4 (2.3) |  | Ref | Ref | 16 (9.6) |  | Ref | Ref |
| Junior high school | 9 (6.4) |  | 2.904 (0.875-9.640) | 1.870 (0.475-7.374) (P=0.371) | 28 (20.6) |  | 2.443 (1.260-4.737) | 1.433 (0.651-3.158) (P=0.371) |
| Senior high school | 7 (7.4) |  | 3.395 (0.967-11.911) | 2.450 (0.469-12.807) (P=0.288) | 26 (27.7) |  | 3.608 (1.818-7.160) | 1.716 (0.668-4.406) (P=0.262) |
| College and higher | 17 (23.3) |  | 12.904 (4.167-39.963) | 15.816 (2.291-109.071) (P=0.005) | 33 (45.2) |  | 7.767 (3.890-15.506) | 4.711 (1.484-14.954) (P=0.009) |
| **Occupation** |  | 13.911 (P=0.003) |  |  |  | 25.219 (P=0.000) |  |  |
| Farmer | 10 (4.1) |  | Ref | Ref | 32 (13.3) |  | Ref | Ref |
| Public or private sector | 10 (18.2) |  | 5.204 (2.048-13.226) | 1.775 (0.353-8.917) (P=0.486) | 21 (38.9) |  | 4.150 (2.142-8.043) | 1.939 (0.680-5.523) (P=0.215) |
| Freelancer and other work | 9 (9.9) |  | 2.570 (1.009-6.546) | 1.239 (0.399-3.842) (P=0.711) | 22 (25.3) |  | 2.208 (1.200-4.064) | 1.150 (0.555-2.382) (P=0.706) |
| Student, unemployed, retired | 8 (9.0) |  | 2.313 (0.883-6.062) | 0.723 (0.161-3.258) (P=0.673) | 28 (31.8) |  | 3.040 (1.697-5.444) | 1.269 (0.553-2.910) (P=0.575) |
| **Work outside** |  | 7.020 (P=0.008) |  |  |  | 5.979 (P=0.014) |  |  |
| No | 26 (6.3) |  | Ref | Ref | 80 (19.8) |  | Ref | Ref |
| Yes | 11 (15.3) |  | 2.687 (1.263-5.716) | 2.689 (1.075-6.726) (P=0.034) | 23 (32.9) |  | 1.986 (1.139-3.461) | 1.418 (0.748-2.686) (P=0.285) |
| **Residence** |  | 1.593 (P=0.207) |  |  |  | 1.989 (P=0.158) |  |  |
| Rural | 27 (6.9) |  | Ref | Ref | 78 (20.4) |  | Ref | Ref |
| Township | 10 (10.8) |  | 1.634 (0.761-3.506) | 0.424 (0.117-1.537) (P=0.192) | 25 (27.2) |  | 1.458 (0.865-2.458) | 0.530 (0.236-1.194) (P=0.126) |
| **Cohabitant** |  | 1.319 (P=0.517) |  |  |  | 1.920 (P=0.383) |  |  |
| <=3 | 5 (5.1) |  | Ref | Ref | 16 (16.8) |  | Ref | Ref |
| 4to5 | 21 (8.3) |  | 1.684 (0.617-4.598) | 3.732 (1.157-12.049) (P=0.028) | 58 (23.2) |  | 1.496 (0.811-2.760) | 2.718 (1.320-5.596) (P=0.007) |
| >6 | 10 (8.8) |  | 1.795 (0.592-5.445) | 5.972 (1.560-22.828) (P=0.009) | 21 (19.3) |  | 1.184 (0.578-2.428) | 2.804 (1.191-6.600) (P=0.018) |
| **Household income** (1CNY=0.16USD) |  | 4.867 (P=0.182) |  |  |  | 17.769 (P=0.000) |  |  |
| <CNY20000 | 9 (4.9) |  | Ref | Ref | 23 (12.7) |  | Ref | Ref |
| CNY20001-30000 | 9 (9.3) |  | 1.990 (0.763-5.192) | 2.016 (0.682-5.954) (P=0.204) | 28 (30.1) |  | 2.960 (1.588-5.517) | 2.298 (1.139-4.637) (P=0.020) |
| CNY30001-50000 | 5 (6.1) |  | 1.261 (0.409-3.886) | 1.275 (0.356-4.563) (P=0.709) | 16 (19.8) |  | 1.697 (0.842-3.419) | 1.381 (0.633-3.016) (P=0.418) |
| >CNY50000 | 14 (11.3) |  | 2.473 (1.035-5.907) | 0.999 (0.310-3.222) (P=0.998) | 36 (30.3) |  | 2.988 (1.662-5.374) | 1.368 (0.646-2.895) (P=0.413) |
| **Outpatient service** |  | 0.477 (P=0.490) |  |  |  | 1.555 (P=0.212) |  |  |
| No | 27 (7.2) |  | Ref | Ref | 76 (20.5) |  | Ref | Ref |
| Yes | 10 (9.2) |  | 1.306 (0.611-2.790) | 0.887 (0.362-2.171) (P=0.793) | 27 (26.2) |  | 1.377 (0.830-2.284) | 1.230 (0.669-2.259) (P=0.505) |
| **Inpatient service** |  | 12.876 (P=0.000) |  |  |  | 5.966 (P=0.015) |  |  |
| No | 32 (11.3) |  | Ref | Ref | 71 (25.6) |  | Ref | Ref |
| Yes | 5 (2.5) |  | 0.201 (0.077-0.526) | 0.226 (0.074-0.692) (P=0.009) | 32 (16.2) |  | 0.562 (0.353-0.894) | 0.668 (0.374-1.191) (P=0.172) |
| **Smartphone** |  | 6.270 (P=0.043) |  |  |  | 21.154 (P=0.000) |  |  |
| Not own | 5 (3.5) |  | Ref | Ref | 15 (10.8) |  | Ref | Ref |
| Own, without network | 4 (6.0) |  | 1.760 (0.457-6.776) | 1.027 (0.209-5.063) (P=0.973) | 9 (13.8) |  | 1.322 (0.546-3.203) | 1.146 (0.417-3.149) (P=0.792) |
| Own, with network | 28 (10.2) |  | 3.132 (1.182-8.295) | 2.442 (0.662-9.016) (P=0.180) | 79 (29.3) |  | 3.423 (1.885-6.215) | 2.206 (0.993-4.899) (P=0.052) |
| **Personal computer** |  | 10.177 (P=0.006) |  |  |  | 12.168 (P=0.002) |  |  |
| Not own | 13 (4.5) |  | Ref | Ref | 46 (16.3) |  | Ref | Ref |
| Own, without network | 6 (13.0) |  | 3.171 (1.141-8.817) | 1.910 (0.573-6.360) (P=0.293) | 14 (31.8) |  | 2.394 (1.179-4.864) | 1.639 (0.725-3.706) (P=0.236) |
| Own, with network | 18 (12.1) |  | 2.921 (1.390-6.142) | 1.611 (0.540-4.816) (P=0.393) | 43 (29.1) |  | 2.108 (1.311-3.389) | 1.202 (0.608-2.373) (P=0.597) |
| **Telemedicine** | **Utilization (n=468)** | | | | **Willing to use (n=468)** | | | |
|  | **Yes (%)** | **χ² test** | **Crude OR** | **Adjusted OR^a^** | **Yes (%) ^b^** | **χ² test** | **Crude OR** | **Adjusted OR^a^** |
| **Sex** |  | 0.150 (P=0.698) |  |  |  | 0.138 (P=0.710) |  |  |
| Male | 11 (6.3) |  | Ref | Ref | 60 (35.9) |  | Ref | Ref |
| Female | 17 (5.5) |  | 0.866 (0.396-1.893) | 0.841 (0.323-2.188) (P=0.722) | 103 (34.2) |  | 0.928 (0.625-1.379) | 1.008 (0.630-1.613) (P=0.974) |
| **Age group** |  | 5.631 (P=0.131) |  |  |  | 17.967 (P=0.000) |  |  |
| 16-30 | 13 (6.5) |  | Ref | Ref | 80 (41.2) |  | Ref | Ref |
| 31-45 | 12 (8.5) |  | 1.336 (0.591-3.022) | 1.244 (0.397-3.900) (P=0.708) | 54 (39.4) |  | 0.928 (0.594-1.450) | 1.613 (0.907-2.866) (P=0.104) |
| 46-60 | 2 (2.0) |  | 0.294 (0.065-1.327) | 1.074 (0.219-5.270) (P=0.930) | 17 (17.9) |  | 0.311 (0.171-0.566) | 0.927 (0.449-1.912) (P=0.836) |
| >60 ^c^ | 1 (2.4) |  | 0.354 (0.045-2.781) | (Combined with 46-60 group) | 11 (26.8) |  | 0.523 (0.247-1.104) | (Combined with 46-60 group) |
| **Education** |  | 23.651 (P=0.000) |  |  |  | 33.764 (P=0.000) |  |  |
| Primary and lower | 4 (2.3) |  | Ref | Ref | 35 (21.3) |  | Ref | Ref |
| Junior high school | 6 (4.3) |  | 1.909 (0.528-6.901) | 0.736 (0.131-4.137) (P=0.728) | 44 (32.6) |  | 1.787 (1.064-3.002) | 0.899 (0.463-1.745) (P=0.754) |
| Senior high school | 5 (5.3) |  | 2.377 (0.623-9.075) | 0.890 (0.126-6.290) (P=0.907) | 40 (43.0) |  | 2.787 (1.600-4.856) | 0.942 (0.421-2.109) (P=0.884) |
| College and higher | 13 (17.8) |  | 9.198 (2.887-29.307) | 2.435 (0.284-20.905) (P=0.417) | 42 (58.3) |  | 5.166 (2.837-9.405) | 1.385 (0.494-3.888) (P=0.535) |
| **Occupation** |  | 26.846 (P=0.000) |  |  |  | 35.683 (P=0.000) |  |  |
| Farmer | 5 (2.0) |  | Ref | Ref | 56 (23.5) |  | Ref | Ref |
| Public or private sector | 11 (20.0) |  | 12.250 (4.058-36.982) | 4.549 (0.674-30.723) (P=0.120) | 31 (57.4) |  | 4.386 (2.367-8.129) | 2.030 (0.782-5.275) (P=0.146) |
| Freelancer and other work | 6 (6.6) |  | 3.463 (1.030-11.639) | 2.377 (0.528-10.708) (P=0.260) | 30 (34.9) |  | 1.745 (1.022-2.980) | 1.161 (0.609-2.214) (P=0.650) |
| Student, unemployed, retired | 6 (6.7) |  | 3.519 (1.046-11.833) | 2.203 (0.379-12.794) (P=0.379) | 44 (51.2) |  | 3.415 (2.034-5.734) | 2.401 (1.171-4.923) (P=0.017) |
| **Work outside** |  | 2.424 (P=0.119) |  |  |  | 4.297 (P=0.038) |  |  |
| No | 21 (5.1) |  | Ref | Ref | 131 (32.9) |  | Ref | Ref |
| Yes | 7 (9.7) |  | 1.999 (0.817-4.891) | 1.613 (0.536-4.855) (P=0.395) | 32 (45.7) |  | 1.716 (1.026-2.872) | 1.473 (0.811-2.672) (P=0.204) |
| **Residence** |  | 14.241 (P=0.000) |  |  |  | 18.888 (P=0.000) |  |  |
| Rural | 15 (3.8) |  | Ref | Ref | 114 (30.2) |  | Ref | Ref |
| Township | 13 (14.0) |  | 4.121 (1.887-8.998) | 0.839 (0.223-3.161) (P=0.796) | 49 (54.4) |  | 2.757 (1.724-4.409) | 0.993 (0.497-1.986) (P=0.984) |
| **Cohabitant** |  | 3.538 (P=0.171) |  |  |  | 2.697 (P=0.260) |  |  |
| <=3 | 6 (6.1) |  | Ref | Ref | 38 (40.9) |  | Ref | Ref |
| 4to5 | 16 (6.3) |  | 1.035 (0.393-2.726) | 2.212 (0.705-6.938) (P=0.174) | 79 (32.0) |  | 0.680 (0.416-1.113) | 0.953 (0.538-1.689) (P=0.869) |
| >6 | 2 (1.8) |  | 0.282 (0.056-1.431) | 0.741 (0.127-4.328) (P=0.739) | 34 (31.5) |  | 0.664 (0.372-1.186) | 1.310 (0.659-2.604) (P=0.441) |
| **Household income** (1CNY=0.16USD) |  | 13.884 (P=0.003) |  |  |  | 33.541 (P=0.000) |  |  |
| <CNY20000 | 5 (2.7) |  | Ref | Ref | 35 (19.7) |  | Ref | Ref |
| CNY20001-30000 | 6 (6.2) |  | 2.382 (0.708-8.016) | 2.404 (0.580-9.954) (P=0.227) | 34 (36.6) |  | 2.353 (1.343-4.124) | 1.895 (1.022-3.515) (P=0.043) |
| CNY30001-50000 | 2 (2.4) |  | 0.886 (0.168-4.665) | 0.930 (0.148-5.853) (P=0.938) | 34 (43.6) |  | 3.151 (1.763-5.630) | 2.557 (1.338-4.889) (P=0.005) |
| >CNY50000 | 15 (12.1) |  | 4.961 (1.754-14.033) | 2.125 (0.504-8.953) (P=0.304) | 60 (50.4) |  | 4.142 (2.473-6.936) | 2.016 (1.062-3.827) (P=0.032) |
| **Outpatient service** |  | 2.990 (P=0.084) |  |  |  | 2.589 (P=0.108) |  |  |
| No | 18 (4.8) |  | Ref | Ref | 121 (33.0) |  | Ref | Ref |
| Yes | 10 (9.2) |  | 2.010 (0.899-4.492) | 1.917 (0.704-5.228) (P=0.203) | 42 (41.6) |  | 1.446 (0.921-2.272) | 1.320 (0.761-2.289) (P=0.323) |
| **Inpatient service** |  | 4.905 (P=0.027) |  |  |  | 0.024 (P=0.878) |  |  |
| No | 22 (7.7) |  | Ref | Ref | 95 (34.5) |  | Ref | Ref |
| Yes | 6 (3.0) |  | 0.371 (0.148-0.932) | 0.569 (0.176-1.837) (P=0.345) | 68 (35.2) |  | 1.031 (0.701-1.517) | 1.511 (0.926-2.465) (P=0.098) |
| **Smartphone** |  | 5.310 (P=0.070) |  |  |  | 30.650 (P=0.000) |  |  |
| Not own | 3 (2.1) |  | Ref | Ref | 27 (19.4) |  | Ref | Ref |
| Own, without network | 4 (6.0) |  | 2.976 (0.647-13.690) | 1.420 (0.233-8.654) (P=0.703) | 16 (24.6) |  | 1.355 (0.671-2.740) | 1.131 (0.508-2.517) (P=0.764) |
| Own, with network | 21 (7.6) |  | 3.834 (1.124-13.082) | 1.221 (0.269-5.540) (P=0.796) | 120 (45.5) |  | 3.469 (2.135-5.634) | 2.038 (1.060-3.916) (P=0.033) |
| **Personal computer** |  | 15.792 (P=0.000) |  |  |  | 25.666 (P=0.000) |  |  |
| Not own | 9 (3.1) |  | Ref | Ref | 73 (26.0) |  | Ref | Ref |
| Own, without network | 1 (2.2) |  | 0.703 (0.087-5.684) | 0.390 (0.041-3.755) (P=0.415) | 17 (40.5) |  | 1.937 (0.990-3.791) | 1.335 (0.618-2.881) (P=0.463) |
| Own, with network | 18 (12.1) |  | 4.303 (1.883-9.834) | 1.376 (0.364-5.202) (P=0.638) | 73 (50.3) |  | 2.881 (1.891-4.388) | 1.438 (0.805-2.570) (P=0.220) |

a. Adjusted OR was estimated based on a multivariable logistic regression model.

b. Response “Yes” for question of willingness to use included those who have utilized such service before.

c. In regression analysis, subgroup by age “>60” was combined with age group “46-60” due to too few positive observations.

Note: N/A, not available; OR, odd ratio
