# Supplementary material for: A profile of patients’ and doctors’ perceptions, acceptance, and utilization of e-health in a deprived region in southwestern China
Source: PLOS Digit Health. 2023 Apr 25;2(4):e0000238. doi: 10.1371/journal.pdig.0000238 (PMC10129013; doi:10.1371/journal.pdig.0000238)
Supplement: S4 Appendix — (DOCX) [file pdig.0000238.s004.docx]

# S4 Appendix. Doctors’ responses to survey items by types of service and user groups

| Question & Item ^a^ | Doctors of county-level hospitals | | | | Doctors of village health centres or clinics | | |  |
| --- | --- | --- | --- | --- | --- | --- | --- | --- |
|  | Sample size | Use before | Willing to use | Reluctant to use | Sample size | Use before | Not use before |  |
|  |  | Number (%) | Number (%) | Number (%) |  | Number (%) | Number (%) |  |
| **1. Online consultation** | **N=153** | **84 (54.9)** | **38 (24.8)** | **31 (20.3)** | **N=59** | **39 (66.1)** | **20 (33.9)** |  |
| 1.1 Concerned factors (multiple responses) |  |  |  |  |  |  |  |  |
| Ease to use / Technology support |  | 46 (54.8) | 17 (44.7) | 6 (19.4) |  | 30 (76.9) |  |  |
| Safety / Quality |  | 52 (61.9) | 17 (44.7) | 20 (64.5) |  | 19 (48.7) |  |  |
| Financial Incentive |  | 18 (21.4) | 10 (26.3) | 3 (9.7) |  | 16 (41.0) |  |  |
| Policy / Insurance support |  | 22 (26.2) | 10 (26.3) | n.a |  | 23 (59.0) |  |  |
| Medical dispute |  | 34 (40.5) | 11 (28.9) | n.a |  | 22 (56.4) |  |  |
| Privacy |  | 46 (54.8) | 19 (50.0) | n.a |  | 15 (38.5) |  |  |
| Other (over workload) |  | 2 (2.4) | 1 (2.6) | 7 (22.6) |  | 0 (0.0) |  |  |
| Missing data |  | 0 (0.0) | 0 (0) | 0 (0.0) |  | 0 (0.0) |  |  |
| 1.2 Motivation to use (multiple responses) |  |  |  |  |  |  |  |  |
| Work needs (follow-up) |  | 57 (67.9) | 25 (65.8) |  |  | 27 (69.2) |  |  |
| Required by patients |  | 40 (47.6) | 35 (92.1) |  |  | 15 (38.5) |  |  |
| Reputation |  | 14 (16.7) | 7 (18.4) |  |  | 1 (2.6) |  |  |
| Income |  | 16 (19.0) | 6 (15.8) |  |  | 0 (0.0) |  |  |
| Other |  | 2 (2.4) | 1 (2.6) |  |  | 1 (2.6) |  |  |
| Missing data |  | 0 (0.0) | 0 (0) |  |  | 0 (0.0) |  |  |
| 1.3 Manner (multiple responses) |  |  |  |  |  |  |  |  |
| Phone call |  | 78 (92.9) | 20 (52.6) |  |  | 35 (89.7) |  |  |
| Message |  | 41 (48.8) | 7 (18.4) |  |  | 20 (51.3) |  |  |
| WeChat |  | 48 (57.1) | 17 (44.7) |  |  | 18 (46.2) |  |  |
| Official website |  | 17 (20.2) | 21 (55.3) |  |  | 2 (5.1) |  |  |
| Third-party platform |  | 7 (8.3) | 7 (18.4) |  |  | 0 (0.0) |  |  |
| Other |  | 3 (3.6) | 0 (0.0) |  |  | 0 (0.0) |  |  |
| Missing data |  | 0 (0.0) | 0 (0) |  |  | 0 (0.0) |  |  |
| 1.4 Disease (multiple responses) |  |  |  |  |  |  |  |  |
| Chronic disease / General symptoms |  | 70 (83.3) | 32 (84.2) |  |  | 33 (84.6) |  |  |
| Severe disease (surgery / tumour) |  | 32 (38.1) | 2 (5.3) |  |  | 9 (23.1) |  |  |
| Not specified |  | 12 (14.3) | 4 (10.5) |  |  | 0 (0.0) |  |  |
| Missing data |  | 0 (0.0) | 0 (0) |  |  | 0 (0.0) |  |  |
| 1.5 Content (multiple responses) |  |  |  |  |  |  |  |  |
| Diagnosis |  | 59 (70.2) | 15 (39.5) |  |  | 29 (74.4) |  |  |
| Treatment |  | 58 (69.0) | 22 (57.9) |  |  | 22 (56.4) |  |  |
| Fee |  | 48 (57.1) | 7 (18.4) |  |  | 9 (23.1) |  |  |
| Medication |  | 50 (59.5) | 19 (50.0) |  |  | 18 (46.2) |  |  |
| Follow-up |  | 30 (35.7) | 14 (36.8) |  |  | 18 (46.2) |  |  |
| Others |  | 7 (8.3) | 5 (13.2) |  |  | 0 (0.0) |  |  |
| Missing data |  | 0 (0.0) | 0 (0) |  |  | 0 (0.0) |  |  |
| 1.6 Confirm patient's identification |  |  |  |  |  |  |  |  |
| Yes |  | 71 (84.5) | 31 (81.6) |  |  | 25 (64.1) |  |  |
| No |  | 13 (15.5) | 7 (18.4) |  |  | 6 (15.4) |  |  |
| Unclear/Not specified |  | 0 (0.0) | 0 (0.0) |  |  | 8 (20.5) |  |  |
| Missing data |  | 0 (0.0) | 0 (0) |  |  | 0 (0.0) |  |  |
| 1.7 Involving disease diagnosis |  |  |  |  |  |  |  |  |
| Yes |  | 30 (35.7) |  |  |  | 27 (69.2) |  |  |
| No |  | 54 (64.3) |  |  |  | 8 (20.5) |  |  |
| Unclear/Not specified |  | 0 (0.0) |  |  |  | 4 (10.3) |  |  |
| Missing data |  | 0 (0.0) |  |  |  | 0 (0.0) |  |  |
| 1.8 Involving treatment or drug prescription |  |  |  |  |  |  |  |  |
| Yes |  | 18 (21.4) |  |  |  | 12 (30.8) |  |  |
| No |  | 66 (78.6) |  |  |  | 17 (43.6) |  |  |
| Unclear/Not specified |  | 0 (30.9) |  |  |  | 10 (25.6) |  |  |
| Missing data |  | 0 (0.0) |  |  |  | 0 (0.0) |  |  |
| 1.9 Actual payment |  |  |  |  |  |  |  |  |
| Free |  | 81 (93.1) |  |  |  | 37 (94.8) |  |  |
| CNY>0 |  | 3 (6.9) |  |  |  | 2 (5.1) |  |  |
| Unclear/Not specified |  | 0 (0.0) |  |  |  | 0 (0.0) |  |  |
| Missing data |  | 0 (0.0) |  |  |  | 0 (0.0) |  |  |
| 1.10 Expected payment |  |  |  |  |  |  |  |  |
| Free |  | 19 (22.6) | 4 (10.5) |  |  |  |  |  |
| CNY1-10 |  | 31 (36.9) | 18 (47.4) |  |  |  |  |  |
| CNY11-50 |  | 19 (22.6) | 11 (28.9) |  |  |  |  |  |
| CNY>50 |  | 15 (17.9) | 5 (13.2) |  |  |  |  |  |
| Missing data |  | 0 (0.0) | 0 (0) |  |  |  |  |  |
| **2. Telemedicine / Telehealth** | **N=153** | **45 (29.4)** | **80 (52.3)** | **28 (18.3)** | **N=59** | **15 (25.4)** | **44 (74.6)** |  |
| 2.1 Concerned factors (multiple responses) |  |  |  |  |  |  |  |  |
| Ease to use / Technology support |  | 22 (48.9) | 42 (52.5) | 19 (67.9) |  | 9 (60.0) |  |  |
| Safety / Quality |  | 25 (55.6) | 55 (68.8) | 9 (32.1) |  | 6 (40.0) |  |  |
| Financial incentive |  | 6 (13.3) | 9 (11.3) | 1 (3.6) |  | 6 (40.0) |  |  |
| Policy / Insurance support |  | 7 (15.6) | 19 (23.8) | n.a. |  | 3 (20.0) |  |  |
| Medical dispute |  | 6 (13.3) | 28 (35.0) | n.a. |  | 10 (66.7) |  |  |
| Training / Instruction |  | 10 (22.2) | 26 (32.5) | n.a. |  | 11 (73.3) |  |  |
| Other |  | 5 (11.1) | 5 (6.3) | 0 (0.0) |  | 1 (6.7) |  |  |
| Missing data |  | 0 (0.0) | 0 (0.0) | 0 (0.0) |  | 0 (0.0) |  |  |
| 2.2 Perceived advantages (multiple responses) |  |  |  |  |  |  |  |  |
| Increase income |  | 6 (13.3) | 15 (18.8) |  |  | 4 (26.7) |  |  |
| Increase reputation |  | 3 (6.7) | 16 (20.0) |  |  | 2 (13.3) |  |  |
| Improve health outcome |  | 24 (53.3) | 52 (65.0) |  |  | 12 (80.0) |  |  |
| Improve service accessibility |  | 29 (64.4) | 58 (72.5) |  |  | 14 (93.3) |  |  |
| Improve medical skills |  | 18 (40.0) | 42 (52.5) |  |  | 6 (40.0) |  |  |
| Other |  | 3 (6.7) | 7 (8.8) |  |  | 0 (0.0) |  |  |
| Missing data |  | 0 (0.0) | 0 (0.0) |  |  | 0 (0.0) |  |  |
| 2.3 Motivation to use (multiple responses) |  |  |  |  |  |  |  |  |
| Work needs (myself) |  | 29 (64.4) | 38 (47.5) |  |  | 7 (46.7) |  |  |
| Work needs (other hospitals or doctors) |  | 8 (17.8) | 21 (26.3) |  |  | 3 (20.0) |  |  |
| Required by patients |  | 18 (40.0) | 36 (45.0) |  |  | 2 (13.3) |  |  |
| Other (helping others) |  | 12 (26.7) | 3 (3.8) |  |  | 3 (20.0) |  |  |
| Missing data |  | 0 (0.0) | 0 (0.0) |  |  | 0 (0.0) |  |  |
| 2.4 Disease (multiple responses) |  |  |  |  |  |  |  |  |
| Chronic disease / General symptoms |  | 29 (64.4) | 48 (60.0) |  |  | 9 (60.0) |  |  |
| Severe disease (surgery / tumour) |  | 19 (67.9) | 42 (52.5) |  |  | 6 (40.0) |  |  |
| Not specified |  | 4 (14.3) | 2 (2.5) |  |  | 2 (13.3) |  |  |
| Missing data |  | 0 (0.0) | 0 (0.0) |  |  | 0 (0.0) |  |  |
| 2.5 Level of doctor giving instruction |  |  |  |  |  |  |  |  |
| City-level hospital and higher |  | 9 (36.0) |  |  |  | 2 (13.3) |  |  |
| County-level hospital |  | 2 (8.0) |  |  |  | 8 (53.3) |  |  |
| Township and village health centre |  | 0 (0.0) |  |  |  | 11 (73.3) |  |  |
| Other |  | 3 (12.0) |  |  |  | 0 (0.0) |  |  |
| Missing data |  | 11 (44.0) |  |  |  | 0 (0.0) |  |  |
| 2.6 Undertake medical liability |  |  |  |  |  |  |  |  |
| Yes |  | 32 (71.1) |  |  |  | 12 (80.0) |  |  |
| No |  | 1 (2.2) |  |  |  | 2 (13.3) |  |  |
| Unclear/Not specified |  | 12 (26.7) |  |  |  | 1 (6.7) |  |  |
| Missing data |  | 0 (0.0) |  |  |  | 0 (0.0) |  |  |
| 2.7 Sufficient technical support |  |  |  |  |  |  |  |  |
| Yes |  | 32 (71.1) |  |  |  | 12 (80.0) |  |  |
| No |  | 0 (0.0) |  |  |  | 3 (20.0) |  |  |
| Unclear/Not specified |  | 13 (28.9) |  |  |  | 0 (0.0) |  |  |
| Missing data |  | 0 (0.0) |  |  |  | 0 (0.0) |  |  |
| 2.8 Requirement of a doctor at patient’s side |  |  |  |  |  |  |  |  |
| Yes |  |  | 59 (73.8) |  |  |  |  |  |
| No |  |  | 2 (2.5) |  |  |  |  |  |
| Unclear/Not specified |  |  | 19 (23.8) |  |  |  |  |  |
| Missing data |  |  | 0 (0.0) |  |  |  |  |  |
| 2.9 Confirm patient's identification |  |  |  |  |  |  |  |  |
| Yes |  |  | 69 (86.3) |  |  |  |  |  |
| No |  |  | 0 (0.0) |  |  |  |  |  |
| Unclear/Not specified |  |  | 11 (13.8) |  |  |  |  |  |
| Missing data |  |  | 0 (0.0) |  |  |  |  |  |
| 2.10 Face-to-face contact before telemedicine |  |  |  |  |  |  |  |  |
| Yes |  |  | 54 (67.5) |  |  |  |  |  |
| No |  |  | 11 (13.8) |  |  |  |  |  |
| Unclear |  |  | 15 (18.8) |  |  |  |  |  |
| Missing data |  |  | 0 (0.0) |  |  |  |  |  |
| 2.11 Medical history before telemedicine |  |  |  |  |  |  |  |  |
| Yes |  |  | 76 (95.0) |  |  |  |  |  |
| No |  |  | 0 (0.0) |  |  |  |  |  |
| Unclear/Not specified |  |  | 4 (5.0) |  |  |  |  |  |
| Missing data |  |  | 0 (0.0) |  |  |  |  |  |
| 2.12 Patient consent before telemedicine |  |  |  |  |  |  |  |  |
| Yes |  |  | 59 (73.8) |  |  |  |  |  |
| No |  |  | 19 (23.8) |  |  |  |  |  |
| Unclear/Not specified |  |  | 2 (2.5) |  |  |  |  |  |
| Missing data |  |  | 0 (0.0) |  |  |  |  |  |
| 2.13 Physical examination before telemedicine |  |  |  |  |  |  |  |  |
| Yes |  |  | 45 (56.3) |  |  |  |  |  |
| No |  |  | 2 (2.5) |  |  |  |  |  |
| Unclear/Not specified |  |  | 33 (41.3) |  |  |  |  |  |
| Missing data |  |  | 0 (0.0) |  |  |  |  |  |
| 2.14 Specific regulations and standards for telemedicine |  |  |  |  |  |  |  |  |
| Yes |  |  | 65 (81.3) |  |  |  |  |  |
| No |  |  | 4 (5.0) |  |  |  |  |  |
| Unclear/Not specified |  |  | 11 (13.8) |  |  |  |  |  |
| Missing data |  |  | 0 (0.0) |  |  |  |  |  |
| 2.15 Expected payment |  |  |  |  |  |  |  |  |
| Free |  |  | 13 (16.3) |  |  |  |  |  |
| More than normal |  |  | 15 (18.8) |  |  |  |  |  |
| Less than normal |  |  | 4 (5.0) |  |  |  |  |  |
| Same as normal |  |  | 24 (30.0) |  |  |  |  |  |
| Unclear/Not specified |  |  | 24 (30.0) |  |  |  |  |  |
| Missing data |  |  | 0 (0.0) |  |  |  |  |  |
| 2.16 Satisfaction / Performance |  |  |  |  |  |  |  |  |
| Same as normal |  | 15 (33.3) | 26 (32.5) |  |  | 10 (66.7) |  |  |
| Better than normal |  | 22 (48.9) | 2 (2.5) |  |  | 5 (33.3) |  |  |
| Worse than normal |  | 3 (6.7) | 4 (5.0) |  |  | 0 (0.0) |  |  |
| Unclear/Not specified |  | 5 (11.1) | 48 (60.0) |  |  | 0 (0.0) |  |  |
| Missing data |  | 0 (0.0) | 0 (0.0) |  |  | 0 (0.0) |  |  |

a. Questions regarding the willingness to adopt online consultation and telemedicine were only asked for county-level doctors.

Note: n.a., not applicable
