# Supplementary material for: A profile of patients’ and doctors’ perceptions, acceptance, and utilization of e-health in a deprived region in southwestern China
Source: PLOS Digit Health. 2023 Apr 25;2(4):e0000238. doi: 10.1371/journal.pdig.0000238 (PMC10129013; doi:10.1371/journal.pdig.0000238)
Supplement: S5 Appendix — (DOCX) [file pdig.0000238.s005.docx]

# S5 Appendix. Univariate and multivariable analyses of factors associated with doctors’ provision and intention to adopt e-health service

| **Online consultation** | **Provision (n=212)** | | | | **Willing to use (n=153) ^a^** | | | |
| --- | --- | --- | --- | --- | --- | --- | --- | --- |
|  | **Yes (%)** | **χ² test** | **Crude OR** | **Adjusted OR^a^** | **Yes (%) ^c^** | **χ² test** | **Crude OR** | **Adjusted OR^a^** |
| **Type** |  | 2.193 (P=0.139) |  |  |  |  |  |  |
| County-level hospital | 84 (54.9) |  | Ref | Ref |  |  |  |  |
| Village health centre or clinics | 39 (66.1) |  | 1.602 (0.856, 2.996) | 1.264 (0.509, 3.136) (P=0.614) |  |  |  |  |
| **Sex** |  | 1.25 (P=0.264) |  |  |  | 0.123 (P=0.726) |  |  |
| Male | 30 (65.2) |  | Ref | Ref | 23 (82.1) |  | Ref | Ref |
| Female | 93 (56.0) |  | 0.679 (0.344, 1.341) | 0.757 (0.347, 1.65) (P=0.485) | 99 (79.2) |  | 0.83 (0.288, 2.394) | 0.743 (0.206, 2.683) (P=0.650) |
| **Age group ^d^** |  | 5.042 (P=0.08) |  |  |  | 1.366 (P=0.505) |  |  |
| 18-30 | 56 (50.9) |  | Ref | n.a. | 78 (77.2) |  | Ref | n.a. |
| 31-40 | 34 (66.7) |  | 1.932 (0.967, 3.859) |  | 20 (80.0) |  | 1.181 (0.399, 3.495) |  |
| >40 | 21 (67.7) |  | 1.046 (0.404, 2.711) |  | 11 (91.7) |  | 2.762 (0.285, 26.725) |  |
| **Education** |  | 0.263 (P=0.608) |  |  |  | 0.218 (P=0.641) |  |  |
| Secondary school | 40 (60.6) |  | Ref | Ref | 23 (76.7) |  | Ref | Ref |
| Junior college and higher | 83 (56.8) |  | 0.855 (0.473, 1.546) | 0.812 (0.37, 1.782) (P=0.604) | 99 (80.5) |  | 1.254 (0.482, 3.263) | 1.359 (0.42, 4.393) (P=0.608) |
| **Professional rank** |  | 2.225 (P=0.136) |  |  |  | 1.509 (P=0.219) |  |  |
| Junior-grade | 87 (55.1) |  | Ref | Ref | 94 (76.7) |  | Ref | Ref |
| Medium-grade | 36 (66.7) |  | 1.632 (0.847, 3.147) | 1.377 (0.63, 3.013) (P=0.422) | 28 (87.5) |  | 2.126 (0.686, 6.594) | 2.363 (0.583, 9.574) (P=0.229) |
| **Working year** |  | 8.025 (P=0.046) |  |  |  | 1.123 (P=0.771) |  |  |
| 0-3 | 37 (50.0) |  | Ref | Ref | 51 (78.5) |  | Ref | Ref |
| 4-10 | 40 (56.3) |  | 1.288 (0.67, 2.478) | 1.3 (0.618, 2.735) (P=0.489) | 45 (77.6) |  | 0.949 (0.404, 2.23) | 0.856 (0.297, 2.462) (P=0.772) |
| 11-20 | 26 (78.8) |  | 3.717 (1.436, 9.62) | 2.545 (0.85, 7.606) (P=0.095) | 13 (86.7) |  | 1.785 (0.36, 8.86) | 0.741 (0.112, 4.909) (P=0.756) |
| >20 | 19 (61.3) |  | 1.584 (0.674, 3.722) | 1.127 (0.396, 3.216) (P=0.822) | 13 (86.7) |  | 1.785 (0.36, 8.86) | 0.995 (0.138, 7.178) (P=0.996) |
| **Monthly income** (1CNY=0.16USD) |  | 0.072 (P=0.788) |  |  |  | 2.164 (P=0.141) |  |  |
| ≤CNY2,000/US$320 | 53 (57.0) |  | Ref | Ref | 38 (73.1) |  | Ref | Ref |
| >CNY2,000/US$320 | 70 (58.8) |  | 1.077 (0.622, 1.865) | 1.004 (0.451, 2.237) (P=0.991) | 84 (83.2) |  | 1.822 (0.815, 4.074) | 0.996 (0.3, 3.313) (P=0.995) |
| **Expected income** |  | 0.108 (P=0.743) |  |  |  | 0.914 (P=0.339) |  |  |
| ≤CNY6,000/US$960 | 58 (56.9) |  | Ref | Ref | 40 (75.5) |  | Ref | Ref |
| >CNY6,000/US$960 | 65 (59.1) |  | 1.095 (0.634, 1.889) | 1.172 (0.504, 2.726) (P=0.712) | 82 (82.0) |  | 1.478 (0.659, 3.314) | 1.474 (0.43, 5.053) (P=0.537) |
| **Satisfied with incentive system** |  | 0.83 (P=0.362) |  |  |  | 0.54 (P=0.462) |  |  |
| No | 74 (55.6) |  | Ref | Ref | 78 (78.0) |  | Ref | Ref |
| Yes | 49 (62.0) |  | 1.303 (0.738, 2.301) | 1.21 (0.645, 2.275) (P=0.552) | 44 (83.0) |  | 1.377 (0.583, 3.251) | 1.339 (0.494, 3.633) (P=0.566) |
| **Heavy workload** |  | 0 (P=0.987) |  |  |  | 0.099 (P=0.753) |  |  |
| No | 51 (58.0) |  | Ref | Ref | 55 (80.9) |  | Ref | Ref |
| Yes | 72 (58.1) |  | 1.004 (0.577, 1.747) | 0.815 (0.433, 1.536) (P=0.527) | 67 (78.8) |  | 0.878 (0.395, 1.948) | 0.998 (0.402, 2.479) (P=0.997) |
| **Good physical health** |  | 0.075 (P=0.784) |  |  |  | 3.722 (P=0.054) |  |  |
| No | 64 (57.1) |  | Ref | Ref | 59 (73.7) |  | Ref | Ref |
| Yes | 59 (59.0) |  | 1.081 (0.626, 1.868) | 0.425 (0.152, 1.19) (P=0.103) | 63 (86.3) |  | 2.248 (0.978, 5.168) | 1.723 (0.455, 6.534) (P=0.423) |
| **Good mental health** |  | 3.967 (P=0.046) |  |  |  | 5.223 (P=0.022) |  |  |
| No | 48 (50.5) |  | Ref | Ref | 47 (71.2) |  | Ref | Ref |
| Yes | 75 (64.1) |  | 1.75 (1.008, 3.039) | 3.274 (1.169, 9.18) (P=0.024) | 75 (86.2) |  | 2.527 (1.125, 5.677) | 1.713 (0.458, 6.392) (P=0.424) |
| **Smartphone** |  | 2.234 (P=0.327) |  |  |  | 7.425 (P=0.024) |  |  |
| Not own | 7 (41.2) |  | Ref | Ref | 4 (44.4) |  | Ref | Ref |
| Own, without video chat | 36 (61.0) |  | 2.232 (0.744, 6.696) | 2.237 (0.627, 7.988) (P=0.215) | 38 (80.9) |  | 5.304 (1.181, 23.824) | 6.11 (0.933, 40.005) (P=0.059) |
| Own, with video chat | 80 (58.8) |  | 2.037 (0.731, 5.674) | 1.881 (0.577, 6.141) (P=0.294) | 80 (82.5) |  | 5.903 (1.434, 24.306) | 7.553 (1.297, 43.992) (P=0.025) |
| **Personal computer** |  | 7.793 (P=0.02) |  |  |  | 8.025 (P=0.046) |  |  |
| Not own | 7 (53.8) |  | Ref | Ref | 3 (75.0) |  | Ref | Ref |
| Own, without network | 44 (47.8) |  | 0.786 (0.245, 2.52) | 0.664 (0.169, 2.609) (P=0.557) | 64 (80.0) |  | 1.333 (0.13, 13.684) | 0.41 (0.025, 6.639) (P=0.53) |
| Own, with network | 72 (67.3) |  | 1.767 (0.552, 5.654) | 1.305 (0.338, 5.028) (P=0.699) | 55 (79.7) |  | 1.309 (0.126, 13.559) | 0.276 (0.015, 5.018) (P=0.385) |
| **Telemedicine** | **Utilization (n=475)** | | | | **Willing to use (n=475) ^a^** | | | |
|  | **Yes (%)** | **χ² test** | **Crude OR** | **Adjusted OR^a^** | **Yes (%) ^b^** | **χ² test** | **Crude OR** | **Adjusted OR^a^** |
| **Type** |  | 0.157 (P=0.692) |  |  |  |  |  |  |
| County-level hospital | 45 (29.4) | Ref | Ref | Ref |  |  |  |  |
| Village health centre or clinics | 18 (32.2) |  | 1.14 (0.592, 2.198) | 0.941 (0.356, 2.484) (P=0.901) |  |  |  |  |
| **Sex** |  | 0.002 (P=0.967) |  |  |  | 1.319 (P=0.251) |  |  |
| Male | 14 (30.4) |  | Ref | Ref | 25 (89.3) |  | Ref | Ref |
| Female | 50 (30.1) |  | 0.986 (0.485, 2.006) | 1.108 (0.487, 2.524) (P=0.806) | 100 (80.0) |  | 0.479 (0.134, 1.716) | 0.285 (0.059, 1.381) (P=0.119) |
| **Age group ^d^** |  | 1.451 (P=0.484) |  |  |  | 3.282 (P=0.194) |  |  |
| 18-30 | 31 (28.2) |  | Ref | n.a. | 81 (80.2) |  | Ref | n.a. |
| 31-40 | 14 (27.5) |  | 0.966 (0.46, 2.029) |  | 19 (76.0) |  | 0.782 (0.276, 2.212) |  |
| >40 | 12 (38.7) |  | 1.664 (0.644, 4.299) |  | 12 (100.0) |  |  |  |
| **Education** |  | 0.001 (P=0.981) |  |  |  | 0.632 (P=0.427) |  |  |
| Secondary school | 20 (30.3) |  | Ref | Ref | 23 (76.7) |  | Ref | Ref |
| Junior college and higher | 44 (30.1) |  | 0.991 (0.526, 1.865) | 1.467 (0.636, 3.384) (P=0.368) | 102 (82.9) |  | 1.473 (0.56, 3.876) | 0.917 (0.277, 3.031) (P=0.887) |
| **Professional rank** |  | 1.612 (P=0.204) |  |  |  | 2.156 (P=0.142) |  |  |
| Junior-grade | 44 (27.8) |  | Ref | Ref | 96 (79.3) |  | Ref | Ref |
| Medium-grade | 20 (37.0) |  | 1.525 (0.794, 2.93) | 2.282 (1.001, 5.202) (P=0.050) | 29 (90.6) |  | 2.516 (0.708, 8.937) | 3.554 (0.693, 18.229) (P=0.129) |
| **Working year** |  | 4.512 (P=0.211) |  |  |  | 8.025 (P=0.046) |  |  |
| 0-3 | 27 (36.5) |  | Ref | Ref | 50 (76.9) |  | Ref | Ref |
| 4-10 | 16 (22.5) |  | 0.505 (0.243, 1.049) | 0.403 (0.17, 0.955) (P=0.039) | 51 (87.9) |  | 2.182 (0.82, 5.804) | 2.662 (0.809, 8.758) (P=0.107) |
| 11-20 | 9 (27.3) |  | 0.653 (0.265, 1.608) | 0.388 (0.125, 1.204) (P=0.101) | 11 (73.3) |  | 0.825 (0.229, 2.971) | 0.347 (0.063, 1.906) (P=0.223) |
| >20 | 12 (38.7) |  | 1.098 (0.463, 2.606) | 0.754 (0.25, 2.275) (P=0.616) | 13 (86.7) |  | 1.958 (0.397, 9.666) | 0.787 (0.102, 6.11) (P=0.819) |
| **Monthly income** (1CNY=0.16USD) |  | 0.078 (P=0.78) |  |  |  | 2.365 (P=0.124) |  |  |
| ≤CNY2,000/US$320 | 29 (31.2) |  | Ref | Ref | 39 (75.0) |  | Ref | Ref |
| >CNY2,000/US$320 | 35 (29.4) |  | 0.918 (0.509, 1.656) | 1.147 (0.479, 2.748) (P=0.758) | 86 (85.1) |  | 1.904 (0.827, 4.382) | 1.276 (0.349, 4.669) (P=0.713) |
| **Expected income** |  | 0.922 (P=0.337) |  |  |  | 1.022 (P=0.312) |  |  |
| ≤CNY6,000/US$960 | 34 (33.3) |  | Ref | Ref | 41 (77.4) |  | Ref | Ref |
| >CNY6,000/US$960 | 30 (27.3) |  | 0.752 (0.418, 1.354) | 0.514 (0.199, 1.323) (P=0.168) | 84 (84.0) |  | 1.533 (0.664, 3.538) | 0.923 (0.236, 3.604) (P=0.908) |
| **Satisfied with incentive system** |  | 16.558 (P<0.001) |  |  |  | 0.017 (P=0.895) |  |  |
| No | 27 (20.3) |  | Ref | Ref | 82 (82.0) |  | Ref | Ref |
| Yes | 37 (46.8) |  | 3.454 (1.874, 6.365) | 3.951 (2.026, 7.714) (P=0.001) | 43 (81.1) |  | 0.942 (0.4, 2.218) | 0.895 (0.315, 2.545) (P=0.835) |
| **Heavy workload** |  | 0.608 (P=0.436) |  |  |  | 1.058 (P=0.304) |  |  |
| No | 24 (27.3) |  | Ref | Ref | 58 (85.3) |  | Ref | Ref |
| Yes | 40 (32.3) |  | 1.271 (0.696, 2.319) | 1.514 (0.756, 3.037) (P=0.242) | 67 (78.8) |  | 0.641 (0.274, 1.497) | 0.404 (0.151, 1.077) (P=0.070) |
| **Good physical health** |  | 0.43 (P=0.512) |  |  |  | 0.023 (P=0.88) |  |  |
| No | 36 (32.1) |  | Ref | Ref | 65 (81.2) |  | Ref | Ref |
| Yes | 28 (28.0) |  | 0.823 (0.456, 1.484) | 0.774 (0.264, 2.266) (P=0.641) | 60 (82.2) |  | 1.069 (0.47, 2.431) | 0.776 (0.173, 3.487) (P=0.740) |
| **Good mental health** |  | 0.487 (P=0.485) |  |  |  | 0.151 (P=0.697) |  |  |
| No | 31 (32.6) |  | Ref | Ref | 53 (80.3) |  | Ref | Ref |
| Yes | 33 (28.2) |  | 0.812 (0.451, 1.462) | 0.831 (0.283, 2.442) (P=0.736) | 72 (82.8) |  | 1.181 (0.519, 2.69) | 1.363 (0.293, 6.353) (P=0.693) |
| **Smartphone** |  | 0.089 (P=0.957) |  |  |  | 4.518 (P=0.104) |  |  |
| Not own | 5 (29.4) |  | Ref | Ref | 5 (55.6) |  | Ref | Ref |
| Own, without video chat | 17 (28.8) |  | 0.971 (0.297, 3.179) | 1.14 (0.288, 4.513) (P=0.852) | 40 (85.1) |  | 4.561 (0.977, 21.288) | 8.432 (1.212, 58.733) (P=0.031) |
| Own, with video chat | 42 (30.9) |  | 1.074 (0.356, 3.242) | 1.223 (0.333, 4.486) (P=0.762) | 80 (82.5) |  | 3.765 (0.914, 15.5) | 5.888 (1.052, 32.983) (P=0.044) |
| **Personal computer** |  | 3.526 (P=0.172) |  |  |  | 2.339 (P=0.331) |  |  |
| Not own | 2 (15.4) |  | Ref | Ref | 3 (75.0) |  | Ref | Ref |
| Own, without network | 24 (26.1) |  | 1.94 (0.401, 9.39) | 2.406 (0.406, 14.253) (P=0.334) | 62 (77.5) |  | 1.148 (0.112, 11.721) | 0.678 (0.045, 10.237) (P=0.779) |
| Own, with network | 38 (35.5) |  | 3.024 (0.637, 14.357) | 3.6 (0.623, 20.801) (P=0.152) | 60 (87.0) |  | 2.231 (0.209, 23.844) | 1.452 (0.085, 24.754) (P=0.796) |

a. Data on intention to use e-health service were only available for county-hospital doctors (n=153).

b. Adjusted OR was estimated based on a multivariable logistic regression model.

c. Response “Yes” for question of willingness to use included those who have utilized such service before.

d. In regression analysis, age group was not included due to a number of missing data (n=20 of 212, 10.4%)
