# Supplementary material for: A profile of patients’ and doctors’ perceptions, acceptance, and utilization of e-health in a deprived region in southwestern China
Source: PLOS Digit Health. 2023 Apr 25;2(4):e0000238. doi: 10.1371/journal.pdig.0000238 (PMC10129013; doi:10.1371/journal.pdig.0000238)
Supplement: S6 Appendix — (DOCX) [file pdig.0000238.s006.docx]

# S6 Appendix. Weighting patient sample by population demographics

| Variable | Sample (before weighting) | | Sample (after weighing) | | Total population^a^ | |
| --- | --- | --- | --- | --- | --- | --- |
|  | Number | % | Number | % | Number | % |
| Total number | 485 | 100 | 485 | 100 | 146259 | 100 |
| Gender |  |  |  |  |  |  |
| Male | 174 | 35.9 | 248 | 51.1 | 74759 | 51.1 |
| Female | 311 | 64.1 | 237 | 48.9 | 71500 | 48.9 |
| Age |  |  |  |  |  |  |
| 16-60 | 443 | 91.3 | 391 | 80.7 | 118100 | 80.7 |
| >60 | 42 | 8.7 | 93 | 19.3 | 28159 | 19.3 |
| Education level |  |  |  |  |  |  |
| Primary or lower | 176 | 36.3 | 227 | 47.8 | 69872 | 47.8 |
| Junior high | 141 | 29.1 | 169 | 34.9 | 51038 | 34.9 |
| Senior high | 95 | 19.6 | 44 | 9.1 | 13307 | 9.1 |
| College | 73 | 15.1 | 40 | 8.2 | 12042 | 8.2 |
| Any e-health service |  |  |  |  |  |  |
| Use before | 145 | 29.9 | 131 | 27.0 |  |  |
| Willing to use | 148 | 30.5 | 138 | 28.5 |  |  |
| Reluctant to use | 192 | 39.6 | 216 | 44.5 |  |  |
| E-appointment |  |  |  |  |  |  |
| Use before | 60 | 12.4 | 50 | 10.3 |  |  |
| Willing to use | 147 | 30.3 | 139 | 28.7 |  |  |
| Reluctant to use | 278 | 57.3 | 296 | 61.0 |  |  |
| Online consultation |  |  |  |  |  |  |
| Use before | 87 | 18.3 | 77 | 16.3 |  |  |
| Willing to use | 132 | 27.8 | 119 | 25.2 |  |  |
| Reluctant to use | 256 | 53.9 | 277 | 58.6 |  |  |
| Online drug purchase |  |  |  |  |  |  |
| Use before | 37 | 7.8 | 28 | 5.9 |  |  |
| Willing to use | 66 | 13.9 | 58 | 12.3 |  |  |
| Reluctant to use | 371 | 78.3 | 387 | 81.8 |  |  |
| Telemedicine |  |  |  |  |  |  |
| Use before | 28 | 6.0 | 23 | 4.9 |  |  |
| Willing to use | 135 | 28.8 | 124 | 26.6 |  |  |
| Reluctant to use | 305 | 65.2 | 321 | 68.7 |  |  |

a. Population demographics were retrieved from Population Statistics 2020 at local level.
