# Supplementary material for: A profile of patients’ and doctors’ perceptions, acceptance, and utilization of e-health in a deprived region in southwestern China
Source: PLOS Digit Health. 2023 Apr 25;2(4):e0000238. doi: 10.1371/journal.pdig.0000238 (PMC10129013; doi:10.1371/journal.pdig.0000238)
Supplement: S7 Appendix — (DOCX) [file pdig.0000238.s007.docx]

# S7 Appendix. Exclusion of participants recruited from towns or villages

| Variable | Sample (Including all) | | Sample (After exclusion, n=75) | |
| --- | --- | --- | --- | --- |
|  | Number | % | Number | % |
| Total number | 485 | 100 | 410 | 100 |
| Any e-health service |  |  |  |  |
| Use before | 145 | 29.9 | 122 | 29.8 |
| Willing to use | 148 | 30.5 | 135 | 32.9 |
| Reluctant to use | 192 | 39.6 | 153 | 37.3 |
| E-appointment |  |  |  |  |
| Use before | 60 | 12.4 | 51 | 12.4 |
| Willing to use | 147 | 30.3 | 133 | 32.4 |
| Reluctant to use | 278 | 57.3 | 226 | 55.1 |
| Online consultation |  |  |  |  |
| Use before | 87 | 18.3 | 76 | 18.5 |
| Willing to use | 132 | 27.8 | 113 | 27.6 |
| Reluctant to use | 256 | 53.9 | 221 | 53.9 |
| Online drug purchase |  |  |  |  |
| Use before | 37 | 7.8 | 28 | 6.8 |
| Willing to use | 66 | 13.9 | 60 | 14.6 |
| Reluctant to use | 371 | 78.3 | 322 | 78.5 |
| Telemedicine |  |  |  |  |
| Use before | 28 | 6.0 | 24 | 5.9 |
| Willing to use | 135 | 28.8 | 121 | 29.5 |
| Reluctant to use | 305 | 65.2 | 265 | 64.6 |
